# OTUD1 Activates Caspase‐Independent and Caspase‐Dependent Apoptosis by Promoting AIF Nuclear Translocation and MCL1 Degradation

**DOI:** 10.1002/advs.202002874

**Published:** 2021-02-08

**Authors:** Qingyu Luo, Xiaowei Wu, Pengfei Zhao, Yabing Nan, Wan Chang, Xiaolin Zhu, Dan Su, Zhihua Liu

**Affiliations:** ^1^ State Key Laboratory of Molecular Oncology National Cancer Center/National Clinical Research Center for Cancer/Cancer Hospital Chinese Academy of Medical Sciences and Peking Union Medical College Beijing 100021 China; ^2^ Department of Pathology Zhejiang Cancer Hospital Zhejiang 310022 China

**Keywords:** AIF, apoptosis, chemoresistance, deubiquitination, MCL1, OTUD1, oxidative phosphorylation

## Abstract

Apoptosis‐inducing factor (AIF) plays a dual role in regulating cell survival and apoptosis, acting as a prosurvival factor in mitochondria via its NADH oxidoreductase activity and activating the caspase‐independent apoptotic pathway (i.e., parthanatos) after nuclear translocation. However, whether one factor conjunctively controls the separated functions of AIF is not clear. Here, it is shown that OTU deubiquitinase 1 (OTUD1) acts as a link between the two functions of AIF via deubiquitination events. Deubiquitination of AIF at K244 disrupts the normal mitochondrial structure and compromises oxidative phosphorylation, and deubiquitination of AIF at K255 enhances its DNA‐binding ability to promote parthanatos. Moreover, OTUD1 stabilizes DDB1 and CUL4 associated factor 10 (DCAF10) and recruits the cullin 4A (CUL4A)‐damage specific DNA binding protein 1 (DDB1) complex to promote myeloid cell leukemia sequence 1 (MCL1) degradation, thereby activating caspase‐dependent apoptotic signaling. Collectively, these results reveal the central role of OTUD1 in activating both caspase‐independent and caspase‐dependent apoptotic signaling and propose decreased OTUD1 expression as a key event promoting chemoresistance in esophageal squamous cell carcinoma.

## Graphical abstract

This study reveals OTU deubiquitinase 1 (OTUD1) to be a deubiquitinase modulating apoptosis‐inducing factor (AIF) activity. OTUD1 deubiquitinates AIF at K244 to compromise OXPHOS and reduce cell viability. On the other hand, K255 deubiquitination enhances the DNA binding ability of AIF and promotes parthanatos. OTUD1 also recruits CUL4A‐DDB1 complex by stabilizing DCAF10, promotes MCL1 degradation, and activates caspase‐dependent apoptosis pathway.

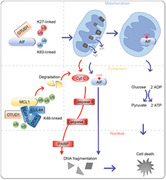

## 1 Introduction

Esophageal cancer ranked seventh in terms of incidence (572 000 new cases) and sixth in overall mortality (509 000 deaths) in 2018, which indicates that esophageal cancer is responsible for an estimated 1 in every 20 cancer deaths.^[^ ^1^ ^]^ Esophageal squamous cell carcinoma (ESCC) is the major histopathological type of esophageal carcinoma, and it accounted for 90% of all cases, with an estimated 478 000 new cases and 375 000 deaths from ESCC in China in 2015.^[^ ^2^ ^]^ Despite advances in treatment, ESCC remains an aggressive tumor with a 5‐year survival rate ranging from 26.2% to 49.4%.^[^ ^3^ ^]^ The combination of cisplatin (DDP) and 5‐fluorouracil is widely used as a standard regimen for ESCC patients; however, whether patients truly benefit from chemotherapy remains controversial.^[^ ^4^, ^5^, ^6^ ^]^ Escape from apoptosis, which endows cancer cells with resistance to chemotherapy, is one of the hallmarks of cancer.^[^ ^7^ ^]^ Therefore, understanding the mechanism underlying the dysregulation of apoptosis in ESCC will reveal previously unknown causes of its chemoresistant characteristics and aid the development of more effective treatment strategies.

Ubiquitination is a common and reversible posttranslational modification that participates extensively in regulating nearly all cellular activities. The ubiquitination reaction is carried out by a three‐step enzymatic catalysis process involving ubiquitin‐activating enzymes (E1), ubiquitin‐conjugating enzymes (E2) and ubiquitin ligases (E3). The E3 ligases determine the specificity of this reaction and the types of ubiquitin linkages (via M1, K6, K11, K27, K29, K33, K48, and K63) on substrates, thus decide the fate of the substrates.^[^ ^8^ ^]^ The K48‐linked ubiquitin chain is the prominent linkage; proteins modified with such ubiquitin chains are commonly targeted to the 26S proteasome for degradation. Compared with the K48‐linked linkage, other linkage types are less studied and associated mostly with nondegradative functions. For example, K6‐linked ubiquitin chains participate in DNA damage repair.^[^ ^9^ ^]^ K11‐linked ubiquitin chains are associated with endoplasmic reticulum‐mediated proteolysis and cell cycle control.^[^ ^10^, ^11^ ^]^ K27‐linked ubiquitin chains exert a critical role in innate immune and inflammatory responses.^[^ ^12^ ^]^ K29‐ or K33‐linked chains are involved in regulating enzyme activities.^[^ ^13^ ^]^ K63‐linked chains are the most studied nondegradative ubiquitin chains that regulate protein kinase activation, the DNA damage response and NF‐*κ*B signaling.^[^ ^14^ ^]^ The process of ubiquitination can be edited and reversed by deubiquitinases (DUBs). To date, more than 600 E3s and approximately 100 DUBs have been discovered to be encoded in the human genome.^[^ ^15^ ^]^ The much smaller number of DUBs than E3s broadens the role of DUBs; in other words, a DUB can exert various functions in different contexts by modulating different substrates. Dysregulation of ubiquitination is associated with various human diseases, especially cancer, and is therefore an attractive and promising clinical therapeutic target.^[^ ^15^, ^16^ ^]^ Although an increasing number of studies have revealed several important roles of DUBs in cancer development, many aspects remain unexplored, especially for DUBs outside of the ubiquitin‐specific peptidase (USP) family.

AIF was named for its ability to induce caspase‐independent apoptosis. The proapoptotic function of AIF results from its nuclear translocation, nonspecific binding to DNA and effect on chromosome condensation and DNA fragmentation.^[^ ^17^ ^]^ Interestingly, the proapoptotic effect of AIF only occurs after its nuclear translocation following an apoptotic stimulus. However, under normal conditions, AIF plays an opposite role, maintaining oxidative phosphorylation (OXPHOS) via the posttranscriptional regulation of major respiratory chain complexes through its NADH oxidoreductase activity in mitochondria.^[^ ^18^, ^19^, ^20^ ^]^ Because of the completely opposing functions of AIF in mitochondria and nuclei, extensive efforts were made to study these two processes separately for decades.^[^ ^21^, ^22^ ^]^ However, whether and how the two functional roles of AIF could be unified remains unrecognized.

Previously, in vitro studies have shown that ubiquitination of AIF might suppress its DNA‐binding ability,^[^ ^23^, ^24^, ^25^ ^]^ yet we do not know the effect of AIF ubiquitination in vivo, especially on chemoresistance in cancer. Moreover, whether the deubiquitination of AIF is sufficient to promote its proapoptotic functions and whether ubiquitination affects the functions of AIF in mitochondria are unclear. Here, via an in vivo screening, we first identified OTUD1 as a proapoptotic DUB in ESCC cells. OTUD1 is also consistently downregulated in DDP‐ and paclitaxel (PTX)‐resistant cell lines compared with their parental line. Mechanistically, we found that OTUD1 directly associates with and deubiquitinates AIF in an atypical manner and that overexpression of OTUD1 is sufficient to promote AIF nuclear translocation and activate caspase‐independent apoptosis. Mass spectrometry (MS) analysis revealed K244 and K255 to be the essential lysine sites on AIF that are modified by OTUD1: K244 deubiquitination leads to the compromise of mitochondrial structure and OXPHOS, while K255 deubiquitination promotes the binding of AIF to DNA. Interestingly, we found that OTUD1 can also promote MCL1 degradation via deubiquitination and stabilization of DCAF10 and that MCL1 degradation provides a stimulus for the release of AIF from mitochondria. The decreased MCL1 protein level also leads to the activation of caspase‐dependent apoptotic signaling, enabling the proapoptotic function of OTUD1 even in the absence of AIF. Our results not only reveal OTUD1 to be a dynamic linker that conjunctively controls the roles of AIF in mitochondria and in the nuclei but also illustrate the central role of OTUD1 in regulating both caspase‐independent and caspase‐dependent apoptotic signaling.

## 2 Results

### 2.1 Low OTUD1 Expression Correlates with Chemoresistance and Poor Prognosis in ESCC

**Figure 1:**
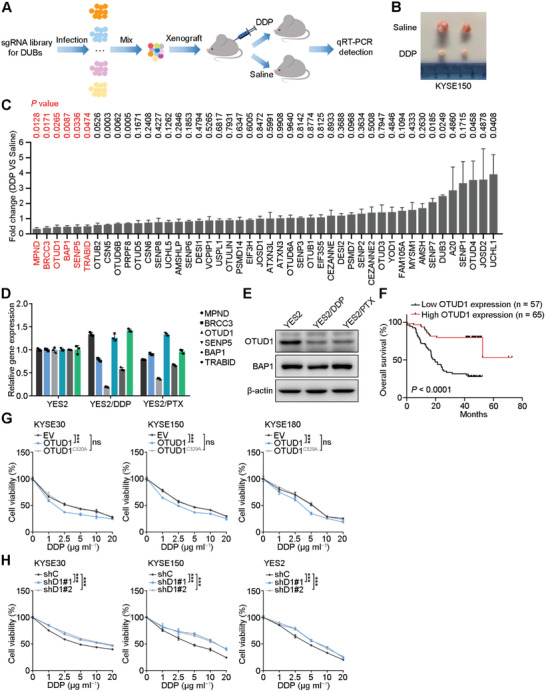
Low OTUD1 expression correlates with chemoresistance and poor prognosis. A) Schematic showing the in vivo screening strategy for proapoptotic DUBs. B) Representative image of xenografts treated with saline or DDP. C) qRT‐PCR analysis for fold change of gene expression of 46 DUBs between DDP‐ and saline‐treated xenografts. The data are the means ± s.e.m.; *n* = 4. Two‐tailed *t* tests. D,E) qRT‐PCR and Western blotting analyses for gene expression in chemoresistant and parental YES2 cell lines. The data are the means ± s.d.; *n* = 3. F) Correlations between OTUD1 expression and overall survival in ESCC patients. *n* = 122. Kaplan‐Meier survival plots are shown. G) Relative viability of ESCC cells expressing empty vector (EV), OTUD1 or OTUD1^C320A^. The data are the means ± s.d.; *n* = 5. Two‐way ANOVA test, ***p < 0.001, ns: not significant. H) Relative viability of ESCC cells expressing OTUD1‐targeting (shD1#1, shD1#2) or nontargeting (shC) shRNAs. The data are the means ± s.d.; *n* = 5. Two‐way ANOVA test, ***p < 0.001.

To identify novel DUBs that play essential roles in chemotherapy resistance in ESCC, we designed an sgRNA library targeting 46 DUBs belonging to the SENP, OTU, MJD, JAMM and UCH families (excluding the relatively well‐studied USP family). KYSE150 cells were plated into 46 plates, infected with viruses harboring these sgRNA sequences separately and then selected with puromycin for 10 days. We next mixed equal amounts of KYSE150 cells infected with viruses harboring the different sgRNA sequences and xenografted the mixed cells subcutaneously into nude mice. One week after xenografting, the mice were divided into two groups and administered DDP or saline for another two weeks before sacrifice (**Figure** **1**A). At the time of sacrifice, the tumors in mice treated with DDP were significantly smaller than those in mice treated with saline (Figure 1B; Figure S1A, Supporting Information). We then extracted RNAs from those xenografts and detected the mRNA expression of 46 DUBs. We focused on the six significantly downregulated DUBs in the DDP‐treated group (Figure 1C) because downregulation of these DUBs may be due to the survival of DUB knockout cells. To further validate the potential role of these six DUBs in chemoresistance, we measured their expression levels in another two in vitro models of chemoresistant ESCC cell lines. More specifically, we induced the ESCC cell line YES2 with DDP and PTX to establish the chemoresistant lines YES2/DDP and YES2/PTX, respectively (Figure S1B, Supporting Information). The qRT‐PCR results showed that only OTUD1 was significantly downregulated in both chemoresistant cell lines compared to the parental YES2 cell line (Figure 1D), and the IB assay results also confirmed a decrease in the OTUD1 protein level (Figure 1E).

We next performed immunohistochemical (IHC) staining of OTUD1 in 122 ESCC samples and found that low OTUD1 expression in ESCC patients significantly correlated with poor prognosis (Figure 1F; Figure S1C,D, Supporting Information), which further supported our speculation that low OTUD1 expression contributes to the chemoresistance and poor prognosis of ESCC patients. To confirm the role of OTUD1 in ESCC chemoresistance, wild‐type OTUD1 or its inactivated mutant counterpart OTUD1^C320A^ was ectopically expressed in KYSE30, KYSE150 and KYSE180 cells, followed by DDP treatment. Overexpression of OTUD1 increased the sensitivity of ESCC cells to DDP treatment, and the mutant lost this ability, which demonstrated that the tumor‐suppressive effect of OTUD1 is dependent on its enzymatic activity (Figure 1G; Figure S1E, Supporting Information). Consistent with this result, OTUD1 depletion using two independent shRNAs markedly increased the chemoresistance of KYSE30, KYSE150, and YES2 cell lines to DDP (Figure 1H; Figure S1F, Supporting Information).

### 2.2 OTUD1 Activates the Caspase‐Independent Apoptosis Pathway

**Figure 2:**
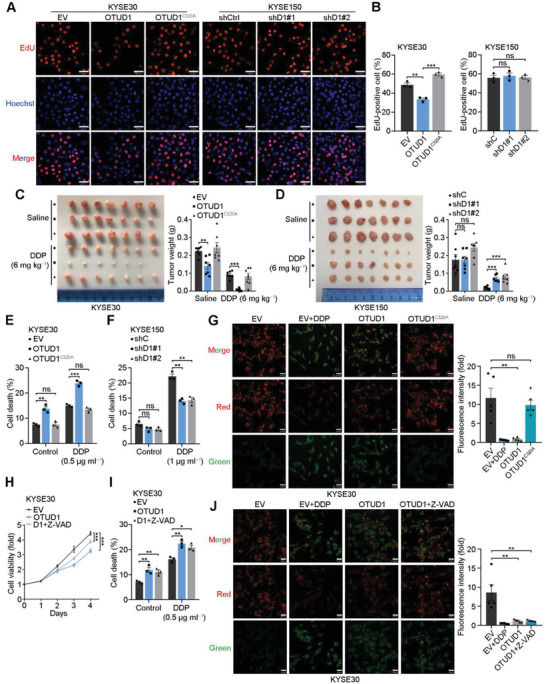
OTUD1 activates the caspase‐independent apoptotic pathway. A,B) Representative images and fraction of EdU‐positive cells. Scale bars, 60 µm. The data are the means ± s.e.m.; *n*  =  3. Two‐tailed *t* tests, ***p < 0.001, **p < 0.01, ns: not significant. C) Representative image and tumor weights of xenografts from KYSE30 cells expressing EV, OTUD1, or OTUD1^C320A^ and treated with saline or DDP. The data are the means ± s.e.m.; *n* = 7. Two‐tailed *t* tests, ***p < 0.001, **p < 0.01. D) Representative image and tumor weights of xenografts from KYSE150 cells expressing shC, shD1#1, or shD1#2 shRNAs and treated with saline or DDP. The data are the means ± s.e.m.; *n* = 7. Two‐tailed *t* tests, ***p < 0.001, ns: not significant. E,F) Statistical analyses of the cell death fraction of the indicated cells as determined using flow cytometric analysis. The data are the means ± s.e.m.; *n*  =  3. Two‐tailed *t* tests, ***p < 0.001, **p < 0.01, ns: not significant. G) Representative image and statistical analyses of JC‐1 staining of KYSE30 cells expressing EV, OTUD1 or OTUD1^C320A^ and treated with DDP. Scale bars, 30 µm. Fluorescence intensity (fold) indicates the relative intensity of Red/Green. The data are the means ± s.e.m.; *n*  =  5. Two‐tailed *t* tests, **p < 0.01, ns: not significant. H) In vitro growth of KYSE30 cells expressing EV or OTUD1 and treated with or without Z‐VAD (50 × 10^−6^ m). The data are the means ± s.d.; *n* = 5. Two‐tailed *t* tests, ***p < 0.001. I) Cell death fractions of the indicated KYSE30 cells expressing EV or OTUD1 and treated with DDP together with or without Z‐VAD (50 × 10^−6^ m). The data are the means ± s.e.m.; *n*  =  3. Two‐tailed *t* tests, **p < 0.01, *p < 0.05. J) Representative image and statistical analyses of JC‐1 staining of KYSE30 cells expressing EV or OTUD1 and treated with DDP or Z‐VAD (50 × 10^−6^ m). Scale bars, 30 µm. Fluorescence intensity (fold) indicates the relative intensity of Red/Green. The data are the means ± s.e.m.; *n*  =  5. Two‐tailed *t* tests, **p < 0.01.

In vitro proliferation assays showed that wild‐type OTUD1, but not the inactivated mutant OTUD1^C320A^, significantly reduced the growth rate of ESCC cells (Figure S2A, Supporting Information). Surprisingly, OTUD1 downregulation in all three ESCC cell lines did not obviously affect their growth rates (Figure S2B, Supporting Information). These results were further confirmed by an EdU staining assay, which indicated that OTUD1 suppresses ESCC cell growth, but OTUD1 depletion cannot promote the growth rates of ESCC cells (**Figure** **2**A,B). The results of in vivo xenograft assays also showed that OTUD1 inhibited ESCC growth and increased chemosensitivity to DDP (Figure 2C). In contrast, OTUD1 depletion increased the chemoresistance of ESCC xenografts to DDP but did not affect tumor growth (Figure 2D). To exclude potential overphysiological effects triggered by ectopic expression, we mutated endogenous OTUD1 into OTUD1^C320A^ in KYSE150 cells and confirmed the role of OTUD1 in promoting chemosensitivity (Figure S2C, Supporting Information).

To explain the controversial role of OTUD1 overexpression and depletion on the growth of ESCC cells, we speculated that the inhibition of cell growth by OTUD1 might result from increased apoptosis rather than suppressed proliferation. To confirm our speculation, annexin V‐FITC/PI double staining assays were performed, and the results showed that OTUD1 overexpression increased the apoptosis rate of KYSE30 cells and promoted their sensitivity to DDP treatment (Figure 2E; Figure S2D, Supporting Information). On the other hand, OTUD1 depletion decreased the apoptotic fraction of DDP‐treated KYSE150 cells (Figure 2F; Figure S2E, Supporting Information). To further evaluate the effect of OTUD1 in the early stage of apoptosis, we used the metachromatic fluorochrome 5,5′,6,6′‐tetrachloro‐1,1′,3,3′‐tetraethylbenzimidazolcarbocyanine iodide (JC‐1) to detect changes in the mitochondrial membrane potential. Overexpression of OTUD1 resulted in a significant decrease in the mitochondrial membrane potential and depolarized the mitochondrial membrane, thus inducing apoptosis (Figure 2G). Apoptotic events can be separated into caspase‐dependent and caspase‐independent pathways depending on their relying on caspase activation. To specify the apoptosis pathway that mediates the proapoptotic function of OTUD1, we treated OTUD1‐overexpressing KYSE30 cells with the pancaspase inhibitor Z‐VAD. All the results showed that Z‐VAD treatment could not completely inhibit the proapoptotic function of OTUD1 (Figure 2H–J; Figure S2F, Supporting Information), indicating that the caspase‐independent pathway is involved in this function.

### 2.3 OTUD1 Interacts with AIF and Promotes AIF Nuclear Translocation

**Figure 3:**
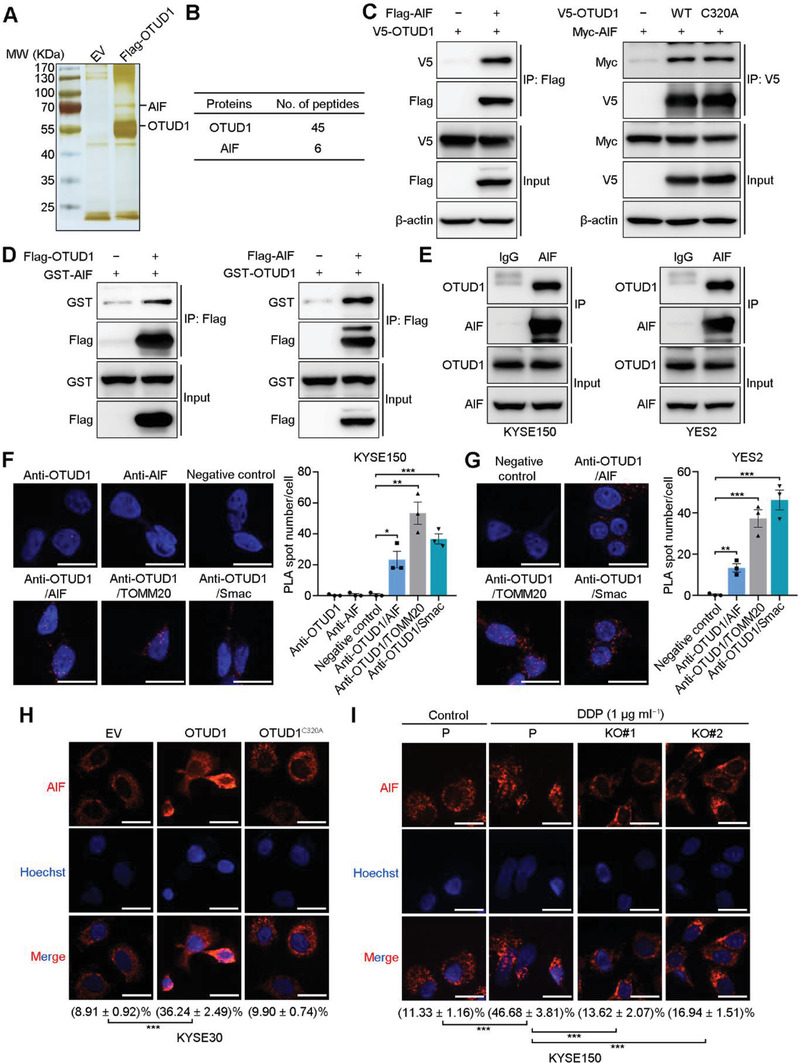
OTUD1 interacts with AIF and promotes its nuclear translocation. A) Silver staining for Flag‐OTUD1‐associated proteins after immunoprecipitation using anti‐Flag magnetic beads. B) Number of unique peptide hits for OTUD1 and AIF. C) IB for the indicated proteins in in vivo co‐IP assays using anti‐Flag and anti‐V5 magnetic beads. D) IB for the indicated proteins in in vitro co‐IP assays using anti‐Flag magnetic beads. E) IB for the indicated proteins in a co‐IP assay using anti‐AIF antibody. F,G) Confocal microscopy assessment of PLA spots (red) in the indicated cells. Each spot indicates a single interaction between proteins. Nuclei were stained with DAPI (blue). Scale bars, 30 µm. The data are the means ± s.e.m.; *n*  =  3. Two‐tailed *t* tests, ***p < 0.001, **p < 0.01, *p < 0.05. H,I) Confocal microscopy assessment of AIF (red) localization in the indicated cells. Nuclei were stained with Hoechst 33 342 (blue). Percentages indicate the relative intensity of the AIF signal inside the nucleus compared to the AIF signal of the whole cell. Scale bars, 30 µm. The data are the means ± s.e.m.; *n* = 15. Two‐tailed *t* tests, ***p < 0.001.

To study the mechanism underlying the activation of the caspase‐independent apoptosis pathway by OTUD1, we transfected Flag‐tagged OTUD1 into 293T cells and performed an immunoprecipitation assay using anti‐Flag magnetic beads. The immunoprecipitated proteins were then subjected to MS analysis to identify potential substrates of OTUD1 (**Figure** **3**A). Among the proteins identified by MS, we focused on AIF (Figure 3A,B), which functions as an inducer of the caspase‐independent apoptosis pathway.^[^ ^17^ ^]^ To validate the interaction between OTUD1 and AIF, we first cotransfected Flag‐AIF and V5‐OTUD1 into 293T cells and performed immunoprecipitation using anti‐Flag magnetic beads. IB analysis confirmed the association between Flag‐AIF and V5‐OTUD1 (Figure 3C). Consistent with this result, co‐immunoprecipitation (co‐IP) experiments using lysates from 293T cells transfected with V5‐OTUD1 or V5‐OTUD1^C320A^ and Myc‐AIF also showed an association between V5‐OTUD1 and Myc‐AIF, and the C320A mutant did not affect their association (Figure 3C). We next performed in vitro pulldown assays to investigate whether OTUD1 could directly bind to AIF. GST‐tagged AIF was confirmed to be directly associated with Flag‐OTUD1, and vice versa (Figure 3D). The association between endogenous OTUD1 and AIF was also validated in two ESCC cell lines by co‐IP assays using anti‐AIF antibody (Figure 3E). To further map the association between OTUD1 and AIF, several truncated mutants were constructed (Figure S3A,B, Supporting Information). Co‐IP assays showed that AIF selectively binds to the linker domain of OTUD1 (Figure S3C, Supporting Information), while OTUD1 associates with the NADH binding and Loop domains of AIF (Figure S3D, Supporting Information). To specify the interacting location between OTUD1 and AIF proteins in vivo, we performed proximity ligation assay (PLA) using antibodies against OTUD1, AIF, translocase of outer mitochondrial membrane 20 (TOMM20, an outer membrane mitochondrial protein facing the cytosol) and second mitochondrial activator of caspases (Smac, a protein located in the mitochondrial intermembrane space). The results showed that OTUD1 interacts with all three mitochondrial proteins in both KYSE150 and YES2 cells (Figure 3F,G). The colocalizations between OTUD1 and TOMM20/Smac were further supported by in vivo co‐IP assays (Figure S3E, Supporting Information). Interestingly, a previous study reported that a fraction of AIF proteins are also located on the outer mitochondrial membrane.^[^ ^26^ ^]^ These results highly suggest that OTUD1 interacts with AIF in both the mitochondrial intermembrane space and cytoplasm.

Ubiquitination and deubiquitination events are involved in the regulation of degradative and nondegradative functions. To validate the effect of OTUD1 on AIF, we first transfected increasing amounts of OTUD1 or OTUD1^C320A^ into 293T cells. IB analysis showed that neither wild‐type nor mutant OTUD1 noticeably affected the protein levels of AIF (Figure S3F, Supporting Information). Consistent with this result, neither OTUD1 overexpression in KYSE30 cells nor OTUD1 depletion in KYSE150 cells affected the protein expression levels of AIF (Figure S3G, Supporting Information). Treatment with the ubiquitin‐proteasome inhibitor MG132 showed no effect on the accumulation of AIF protein (Figure S3H, Supporting Information). These results indicated that OTUD1 did not affect the stability of AIF, and AIF is likely not degraded via the ubiquitin‐proteasome pathway. Since the proapoptotic function of AIF relies on its nuclear translocation, we next determined whether OTUD1 affects AIF nuclear translocation during the apoptosis process. The immunofluorescence and IB results showed that overexpression of OTUD1 promotes the nuclear translocation of AIF, even in the absence of DDP treatment, and the inactivated mutant OTUD1^C320A^ lost this ability (Figure 3H; Figure S3I, Supporting Information). On the other hand, DDP treatment induced AIF nuclear translocation in KYSE150 cells, but AIF nuclear translocation was significantly blocked after *OTUD1* loss (Figure 3I; Figure S3J, Supporting Information).

### 2.4 OTUD1 Deubiquitinates AIF at K244 and K255

**Figure 4:**
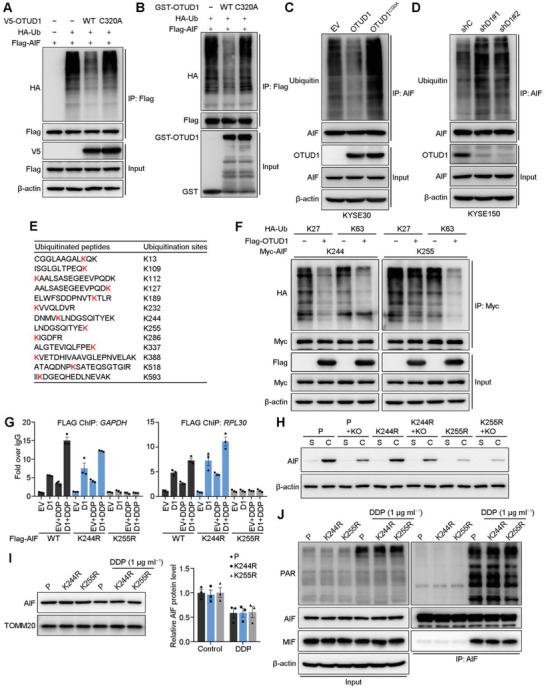
Deubiquitination at AIF K255 leads to an increase in DNA‐binding ability. A) IB to assess AIF ubiquitination in 293T cells cotransfected with Flag‐AIF, HA‐Ub, and V5‐OTUD1 (wild‐type or C320A). B) IB to assess AIF ubiquitination in an in vitro ubiquitination assay. C) IB to assess AIF ubiquitination in KYSE30 cells expressing EV, OTUD1 or OTUD1^C320A^. D) IB detection for the ubiquitination of AIF in KYSE150 cells expressing shC, shD1#1, or shD1#2 shRNAs. E) Ubiquitinated peptides and lysine sites in the MS analysis. F) IB to assess Myc‐AIF ubiquitination in 293T cells cotransfected with Myc‐AIF mutants, HA‐Ub mutants and Flag‐OTUD1. K27, K63, K244, and K255 indicate that all lysines, except K27, K63, K244, or K255, respectively, were mutated to arginines. G) ChIP assays to detect the association between the indicated AIF mutants and genomic DNA of *GAPDH* and *RPL30* in KYSE30 cells. The data are the means ± s.e.m.; *n*  =  3. H) IB detection for the soluble and DNA‐binding AIF mutants in KYSE150 cells. I) IB detection for AIF level in the mitochondria of indicated cells before or after DDP treatment. Quantification of AIF expression relative to TOMM20 expression is shown. The data are the means ± s.e.m.; *n*  =  3. J) IB to assess interactions between AIF and PAR/MIF in the indicated cells.

To study the detailed mechanism by which OTUD1 promotes AIF nuclear translocation, we first asked whether OTUD1 functions as a bona fide DUB of AIF. HA‐Ubiquitin (HA‐Ub), V5‐OTUD1, and Flag‐AIF were cotransfected into 293T cells, and immunoprecipitation was subsequently performed using anti‐Flag magnetic beads. Overexpression of wild‐type OTUD1, but not the inactivated mutant, resulted in significant removal of the ubiquitin chains from AIF (**Figure** **4**A). We next performed an in vitro deubiquitination assay and confirmed that OTUD1 can directly deubiquitinate AIF (Figure 4B). The modulatory effect of OTUD1‐mediated deubiquitination on AIF was also confirmed in ESCC cells (Figure 4C,D). To further specify the lysine site within AIF deubiquitinated by OTUD1, Flag‐AIF was transfected into 293T cells, and immunoprecipitation was performed. The enriched Flag‐AIF proteins were then analyzed for ubiquitinated peptides by MS. A total of 13 ubiquitinated peptides/lysine were detected (Figure 4E). To confirm the key lysine site responsible for OTUD1‐mediated AIF deubiquitination, we individually mutated those 13 lysine residues to arginine and performed deubiquitination assays. The results showed that mutation of either K244 or K255 in AIF significantly reduced the deubiquitination effect of OTUD1, while mutation of the other 11 lysine sites had no such effect (Figure S4A, Supporting Information). To specify the type of polyubiquitin chains on AIF cleaved by OTUD1, a deubiquitination assay was performed using different ubiquitin mutant vectors. The results demonstrated that OTUD1 primarily cleaved the K27‐ and K63‐linked ubiquitin chains (Figure S4B, Supporting Information). To specify the roles of the K244 and K255 sites of AIF in deubiquitination by OTUD1, we constructed mutant forms of AIF with only active K244 or K255 and repeated the deubiquitination assays. OTUD1 consistently cleaved the K27‐ and K63‐linked ubiquitin chains on K244 of AIF but primarily cleaved K63‐linked ubiquitin chains on K255 (Figure 4F). These results revealed that OTUD1 deubiquitinates AIF in an atypical manner and suggested that K244 and K255 within AIF are crucial for its deubiquitination by OTUD1.

### 2.5 Deubiquitination of AIF at K255 Enhances DNA Binding Ability

The proapoptotic function of AIF relies on the long trip from mitochondria to the nucleus, during which it can be affected by many events. Previous studies have shown that different lysine sites in AIF might contribute to different effects on its function.^[^ ^24^ ^]^ To investigate whether K244 and K255 are associated with the proapoptotic role of AIF, we first knocked out endogenous *AIF* in KYSE30 cells and then ectopically expressed wild‐type, K244R, or K255R AIF (AIF^WT^, AIF^K244R^, and AIF^K255R^, respectively). Subcutaneous xenograft assays showed that *AIF* knockout reduced the growth rate of ESCC xenografts (Figure S4C, Supporting Information), which is consistent with the findings of a recent study that revealed the role of AIF in the maintenance of cancer cell survival.^[^ ^22^ ^]^ Moreover, ectopic expression of AIF^WT^ or AIF^K255R^ completely reversed the growth‐suppressive effect, while expression of AIF^K244R^ did not reverse this effect (Figure S4C, Supporting Information), suggesting that K244 was essential for the prosurvival role of AIF in cancer cells.

Previous in vitro studies showed that the ubiquitination of AIF at K255 by X‐linked inhibitor of apoptosis (XIAP) diminished its DNA binding ability and proposed that the decreased DNA binding ability of AIF contributes to the antiapoptotic function of XIAP.^[^ ^23^ ^]^ To further investigate the role of K244 and K255 in the DNA‐binding ability of AIF in vivo, we modified a chromatin immunoprecipitation (ChIP) assay to assess the association between AIF and DNA. Considering the unspecific association mode of AIF and DNA, we ectopically expressed empty vector or V5‐OTUD1, together with Flag‐AIF^WT^, Flag‐AIF^K244R^, or Flag‐AIF^K255R^, to ensure that a sufficient amount of AIF proteins were enriched for detection of DNA binding. Two primer sets targeting the housekeeping genes *GAPDH* and *RPL30* were used in the ChIP‐qPCR analysis. The results showed that the K255R mutation of AIF abolished its DNA binding ability, while the K244R mutation showed no such effect (Figure 4G), which is in accordance with previous studies.^[^ ^23^ ^]^ Moreover, ectopic OTUD1 enhanced the DNA binding ability of AIF, which was further promoted by additional DDP treatment (Figure 4G). We next investigated the roles of K244 and K255 in the DNA binding ability of endogenous AIF. Endogenous AIF in KYSE150 cells was mutated into AIF^K244R^ and AIF^K255R^, respectively, then *OTUD1* was further ablated. After treatment with DDP for 24 h, nuclear proteins were extracted and separated into soluble and chromatin‐associated parts. IB detection for AIF showed that *OTUD1* ablation reduced the DNA binding of wild‐type AIF and AIF^K244R^, but not of AIF^K255R^ (Figure 4H). These results indicated that although both K244 and K255 are critical for the OTUD1‐induced deubiquitination of AIF, only K255 determines the DNA‐binding ability of AIF, and K244 may affect other aspects of the proapoptotic functions of AIF. Consistently, only the K255R mutant, but not the K244R mutant, diminished the ubiquitination effect of XIAP on AIF, which suggests that other E3 ligases contribute to the ubiquitination of AIF at K244 (Figure S4D, Supporting Information).

We lastly investigated whether K255R mutation affects processes other than DNA binding during AIF nuclear translocation. We treated KYSE150 cells expressing endogenous wild‐type AIF, AIF^K244R^, or AIF^K255R^ proteins with DDP and performed subcellular fractionations to isolate the mitochondrial fraction. All three kinds of AIF proteins were similarly enriched in the resting status and equally diminished from mitochondria upon DDP treatment (Figure 4I). The release of AIF from mitochondria during parthanatos is triggered by its association with poly (ADP‐ribose) (PAR).^[^ ^27^ ^]^ After release from mitochondria, AIF recruits macrophage migration inhibitory factor (MIF) to nuclei to mediate DNA fragmentation and subsequent cell death.^[^ ^28^ ^]^ We then asked whether K255R mutation affect the association between AIF and PAR or MIF. Immunoprecipitation assays showed that neither K244R nor K255R mutation affects the association between AIF and PAR or MIF (Figure 4J), which is in consist with a previous study.^[^ ^29^ ^]^

### 2.6 Deubiquitination of AIF at K244 Disrupts Mitochondrial Structure and Compromises OXPHOS

**Figure 5:**
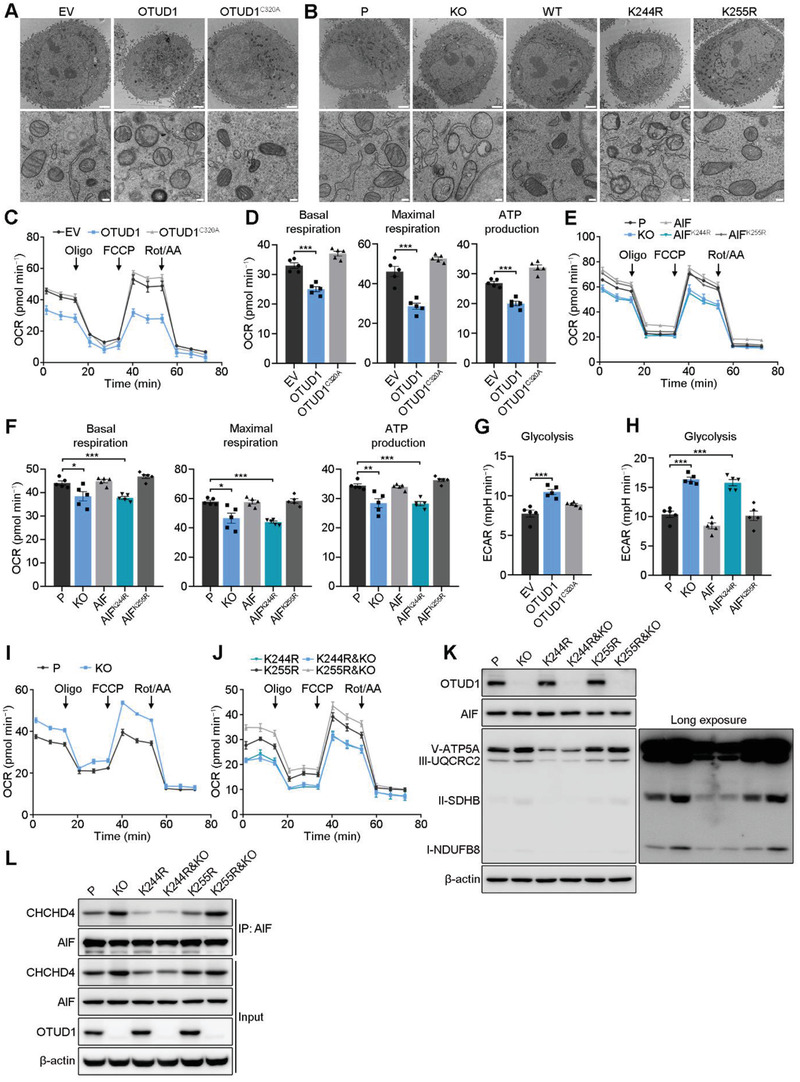
Deubiquitination at AIF K244 disrupts mitochondrial structure and compromises OXPHOS. A,B) Representative electron microscopy images of the indicated cells (KYSE30 background). Scale bars, 2 µm in the top panel and 200 nm in the bottom panel. C–F) Whole‐cell OCR as measured using C,E) Seahorse assays and D,F) statistical analyses of basal respiration, maximal respiration and ATP production in the indicated cells (KYSE30 background). The data are the means ± s.e.m.; *n*  =  5. Two‐tailed *t* tests, ***p < 0.001, **p < 0.01, *p < 0.05. G,H) Statistical analyses of the basal glycolysis rate in the indicated cells (KYSE30 background). The data are the means ± s.e.m.; *n*  =  5. Two‐tailed *t* tests, ***p < 0.001. I,J) Whole‐cell OCR as measured using Seahorse assays in the indicated cells (KYSE150 background). The data are the means ± s.e.m.; *n*  =  5. K) IB for respiratory chain proteins in the indicated cells (KYSE150 background). L) IB to assess interactions between AIF and CHCHD4 in the indicated cells (KYSE150 background).

AIF plays an important role in maintaining the normal structure and function of mitochondria, and previous studies revealed that loss of AIF resulted in abnormal mitochondrial morphology and impaired OXPHOS.^[^ ^20^, ^22^, ^30^ ^]^ To assess the role of OTUD1‐mediated AIF deubiquitination in mitochondrial structure and OXPHOS, we used transmission electron microscopy to visualize the mitochondrial ultrastructure. Overexpression of wild‐type OTUD1, but not OTUD1^C320A^, induced disorder of the mitochondrial structure and resulted in swollen mitochondria without intact cristae (**Figure** **5**A). More importantly, ectopic expression of AIF^WT^ or AIF^K255R^, but not AIF^K244R^, rescued the *AIF* knockout‐induced cristolysis (Figure 5B). These results indicate that K244 of AIF is critical for its role in maintaining the normal structure of mitochondria, and its mutation may promote cell death via compromised OXPHOS.

Mitochondria play an essential role in maintaining OXPHOS to sustain cell survival. Due to the essential role of AIF K244 in maintaining mitochondrial structure, we next investigated whether OTUD1 affects OXPHOS. We first performed a Seahorse assay to assess the oxygen consumption rate (OCR) in KYSE30 cells expressing empty vector, OTUD1 and OTUD1^C320A^. OTUD1‐overexpressing cells consumed oxygen at a significantly lower basal level and produced less ATP than control or OTUD1^C320A^‐overexpressing cells (Figure 5C,D). Upon treatment with the uncoupler FCCP, OTUD1‐overexpressing KYSE30 cells exhibited a limited increase in the OCR, which indicates that their maximal respiratory capacity was reduced (Figure 5C,D). IB analysis showed a reduction in respiratory chain proteins, such as CI‐NDUFB8, CII‐SDHB, CIII‐UQCRC2, and CV‐ATP5A (Figure S5A, Supporting Information), indicating that OTUD1 reduced respiratory chain biogenesis. We next validated whether the compromise of OXPHOS in OTUD1‐overexpressing cells is due to AIF deubiquitination at K244. Endogenous *AIF* was knocked out in KYSE30 cells, and exogenous AIF^WT^, AIF^K244R^, or AIF^K255R^ was then ectopically expressed. Consistent with the results of previous studies,^[^ ^22^, ^31^ ^]^ *AIF* knockout cells exhibited a significantly lower basal OCR and a limited increase in the OCR in response to FCCP (Figure 5E,F). Notably, ectopic expression of AIF^WT^ or AIF^K255R^, but not AIF^K244R^, reversed the disruption of OXPHOS (Figure 5E,F). Consistent with these results, the ectopic expression of only AIF^K244R^ failed to reverse the reduction in respiratory chain proteins (Figure S5B, Supporting Information). To assess whether disrupted OXPHOS promotes glycolysis in ESCC cells, a Seahorse glycolysis stress test was performed. Upon stimulation of glycolysis by glucose addition, the basal extracellular acidification rate (ECAR) was higher in KYSE30 cells expressing OTUD1 than in control cells (Figure 5G). Consistent with this finding, *AIF* knockout cells showed a relatively high ECAR compared to that in parental cells, and ectopic expression of wild‐type AIF, but not AIF^K244R^, reversed the increase in ECAR (Figure 5H).

To further specify that the K244 site of AIF played the key role in the effect of OTUD1 on OXPHOS, we first assessed the OCR and ECAR of wild‐type and *OTUD1*‐knockout KYSE150 cells. Consistent with previous conclusions, *OTUD1* knockout increased OXPHOS and decreased glycolysis in KYSE150 cells (Figure 5I; Figure S5C,D, Supporting Information). We next depleted *OTUD1* in KYSE150 cells with endogenous K244R or K255R mutant AIF and repeated the Seahorse assays. The results showed that *OTUD1* ablation activated OXPHOS in KYSE150 cells with K255R mutant AIF, but not in cells with K244R mutant AIF (Figure 5J; Figure S5E, Supporting Information). Moreover, the cells with the K244R mutant showed no change in respiratory chain proteins in response to *OTUD1* ablation (Figure 5K). Although the ectopic expression of AIF only rescued, but did not elevate, the OXPHOS and respiratory chain proteins (Figure 5E,F; Figure S5B, Supporting Information), the ablation of *OTUD1* significantly increased OXPHOS and respiratory chain proteins (Figure 5I,K; Figure S5C, Supporting Information). These results may be because endogenous AIF has already maintained a sufficient amount for normal functions to maintain cell survival, and the additional AIF could not further promote its functions. However, *OTUD1* ablation enhanced the activity of AIF via deubiquitination modifications to further promote the functions of AIF in mitochondrial respiration. We next explored how OTUD1 affects the OXPHOS and respiratory chain proteins via AIF deubiquitination. The interaction of AIF with coiled‐coil‐helix‐coiled‐coil‐helix domain 4 (CHCHD4) has been suggested to contribute to its role in respiratory chain biogenesis.^[^ ^31^ ^]^ Therefore, we hypothesized that OTUD1 might affect the OXPHOS via interfering with the association between AIF and CHCHD4. We then performed immunoprecipitation using anti‐AIF antibody in KYSE150 cells with endogenous wild‐type, K244R or K255R mutant AIF, with or without *OTUD1* depletion. Consistent with our hypothesis, *OTUD1* depletion promoted the associations between AIF^WT^/AIF^K255R^ and CHCHD4, and K244R mutation nearly abolished the interaction between AIF protein and CHCHD4 (Figure 5L). These results demonstrated a convincing conclusion that OTUD1 compromises OXPHOS and induces a switch in cellular metabolism towards glycolysis via the deubiquitination of AIF at K244.

### 2.7 OTUD1 Recruits the CUL4A‐DDB1 Complex to Promote MCL1 Degradation and Activate the Caspase‐Dependent Apoptosis Pathway

To further validate whether the OTUD1‐induced deubiquitination of AIF is sufficient to promote its nuclear translocation, we performed confocal microscopy to study the localization of AIF with different mutations. Surprisingly, neither the K244R nor K255R AIF mutant underwent nuclear translocation (Figure S6A, Supporting Information). This phenomenon suggests that although OTUD1‐induced deubiquitination of AIF plays an essential role in OXPHOS and promotes the binding of AIF to DNA, other events underlying the promotive effect of OTUD1 on AIF nuclear translocation required further characterization. Another clue that mechanisms other than AIF‐mediated caspase‐independent apoptosis coexist and underlie the proapoptotic function of OTUD1 is that although Z‐VAD treatment could not entirely suppress the proapoptotic role of OTUD1 on ESCC cells, it indeed showed some rescuing effects, which indicates that the caspase‐dependent apoptosis pathway is also involved in the proapoptotic functions of OTUD1 (Figure 2H,I; Figure S2F, Supporting Information). Taken together, the abovementioned evidence drove us to identify an extra factor that may affect the release of AIF and caspase‐dependent apoptosis pathway simultaneously.

**Figure 6:**
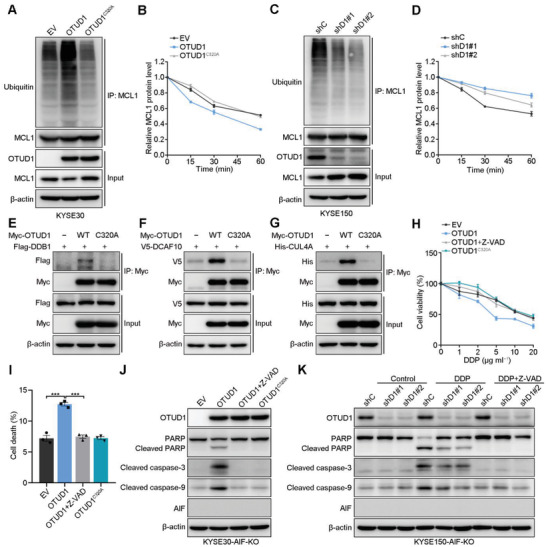
OTUD1 activates the caspase‐dependent apoptotic pathway by recruiting the CUL4A‐DDB1 complex to facilitate ubiquitination‐mediated degradation of MCL1. A) IB to assess MCL1 ubiquitination in KYSE30 cells expressing EV, OTUD1, or OTUD1^C320A^. B) Quantitative analyses of MCL1 expression in KYSE30 cells expressing EV, OTUD1, or OTUD1^C320A^ and subjected to a CHX pulse‐chase assay. Quantification of MCL1 expression relative to *β*‐actin expression is shown. The data are the means ± s.e.m.; *n*  =  3. C) IB to assess MCL1 ubiquitination in KYSE150 cells expressing shC, shD1#1, or shD1#2 shRNAs. D) Quantitative analyses of MCL1 expression in KYSE150 cells expressing shC, shD1#1, or shD1#2 shRNAs and subjected to a CHX pulse‐chase assay. Quantification of MCL1 expression relative to *β*‐actin expression is shown. The data are the means ± s.e.m.; *n*  =  3. E–G) IB for the indicated proteins in co‐IP assays. H) Relative viability of *AIF*‐ablated KYSE30 cells expressing EV, OTUD1, or OTUD1^C320A^ and treated with DDP with or without Z‐VAD (50 × 10^−6^ m). The data are the means ± s.d.; *n* = 5. I) Cell death fraction of *AIF*‐ablated KYSE30 cells expressing EV, OTUD1, or OTUD1^C320A^ and treated with or without Z‐VAD (50 × 10^−6^ m). The data are the means ± s.e.m.; *n*  =  3. Two‐tailed *t* tests, ***p < 0.001. J)IB for the indicated proteins in *AIF*‐ablated KYSE30 cells expressing EV, OTUD1, or OTUD1^C320A^ or in K) *AIF*‐ablated KYSE150 cells expressing shC, shD1#1, or shD1#2 shRNAs and treated with or without DDP (5 µg mL^−1^)/Z‐VAD (50 × 10^−6^ m).

The release of AIF from mitochondria begins with an increase in the permeability of the mitochondrial membrane, which always occurs in response to certain apoptotic stimuli, as exemplified by the downregulation of antiapoptotic BCL‐2 family members.^[^ ^32^, ^33^, ^34^ ^]^ Therefore, we performed IB to measure the expression levels of BCL‐2, BCL‐XL, BCL‐W, and MCL1 in KYSE30 cells expressing empty vector, OTUD1, or OTUD1^C320A^. Only MCL1 was significantly downregulated upon OTUD1 overexpression (Figure S6B, Supporting Information). The qRT‐PCR results showed that the mRNA expression level of MCL1 remained unchanged following OTUD1 overexpression (Figure S6C, Supporting Information), which suggests that OTUD1‐induced downregulation of MCL1 occurs at the posttranslational level. The results of ubiquitination assays and pulse‐chase assays showed that OTUD1 promotes MCL1 ubiquitination and degradation in KYSE30 cells (**Figure** **6**A,B; Figure S6D, Supporting Information). In contrast, OTUD1 depletion decreased the ubiquitination level of endogenous MCL1 and increased its stability (Figure 6C,D; Figure S6E, Supporting Information). To explore the mechanism by which OTUD1 promotes the ubiquitination and degradation of MCL1, we reanalyzed the MS data and found no peptides of MCL1 identified to be associated with OTUD1. An in vitro ubiquitination assay also showed that OTUD1 showed no direct effect on promoting MCL1 ubiquitination (Figure S6F, Supporting Information). These results indicated that the ubiquitination of MCL1 is indirectly mediated by OTUD1. Interestingly, two molecules (DDB1 and DCAF10) in the MS data attracted our attention because DCAFs function as substrate receptors for the CUL4‐DDB1 ubiquitin ligase,^[^ ^35^ ^]^ which was identified as mediating the ubiquitination of MCL1 in a recent study.^[^ ^36^ ^]^ We next performed co‐IP assays and showed that CUL4A, DDB1, and DCAF10 associated with wild‐type OTUD1, but not OTUD1^C320A^, in vivo (Figure 6E–G). Collectively, these results showed that OTUD1 indirectly promotes MCL1 ubiquitination and degradation via recruitment of the CUL4A‐DDB1 complex in a manner that is dependent on its enzymatic activity.

We next evaluated the role of OTUD1 in the context of *AIF* knockout to further validate the role of OTUD1 in caspase‐dependent apoptosis. Overexpression of wild‐type OTUD1 increased the sensitivity of *AIF*‐knockout KYSE30 cells to DDP treatment (Figure 6H,I; Figure S6G, Supporting Information). However, Z‐VAD treatment completely abolished the proapoptotic effect of OTUD1 in *AIF*‐knockout KYSE30 cells (Figure 6H,I; Figure S6G, Supporting Information). IB further confirmed the activation of the caspase‐dependent apoptotic pathway in OTUD1‐overexpressing cells with *AIF* ablation, which was diminished by Z‐VAD (Figure 6J). In contrast, OTUD1 depletion in *AIF*‐knockout KYSE150 cells suppressed the activation of caspase‐dependent apoptotic signaling, and Z‐VAD treatment completely abolished the activation of apoptosis events (Figure 6K). These results showed that OTUD1 can also induce caspase‐dependent apoptotic signaling via the promotion of MCL1 degradation, which is independent of the effects of OTUD1 on AIF.

### 2.8 OTUD1 Deubiquitinates and Stabilizes DCAF10 to Promote MCL1 Ubiquitination and Degradation

**Figure 7:**
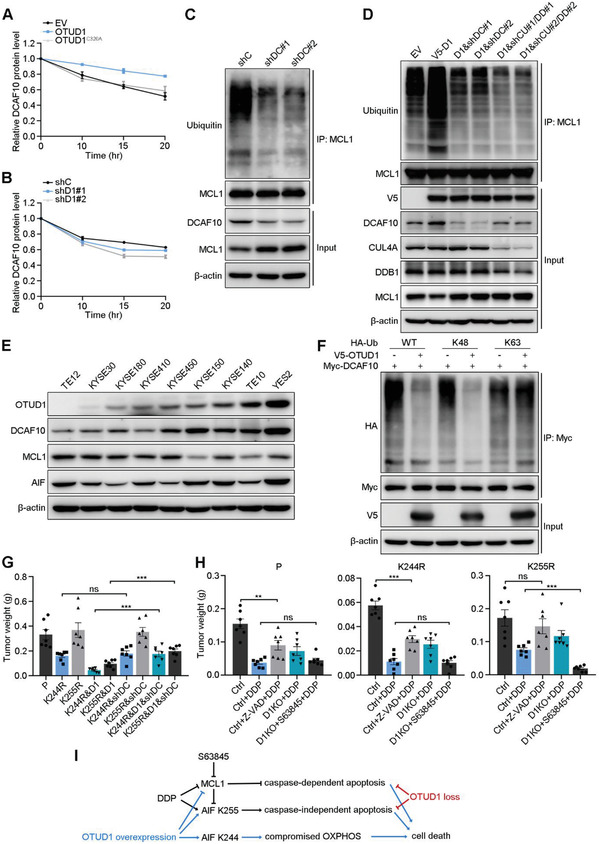
OTUD1 deubiquitinates and stabilizes DCAF10. A,B) Relative protein expression levels of DCAF10 in a pulse‐chase assay performed in the indicated A) KYSE30 or B) KYSE150 cells. Quantification of DCAF10 expression relative to *β*‐actin expression is shown. The data are the means ± s.e.m.; *n*  =  3. C,D) IB to assess MCL1 ubiquitination in the indicated C) KYSE150 or D) KYSE30 cells (D1:OTUD1; shDC: shDCAF10; shCU: shCUL4A; shDD: shDDB1). E) IB for OTUD1, DCAF10, MCL1, and AIF expression in ESCC cell lines. F) IB to assess Myc‐DCAF10 ubiquitination in 293T cells cotransfected with Myc‐DCAF10, HA‐Ub mutants and V5‐OTUD1. K48 and K63 indicate that all lysines except K48 or K63 were mutated to arginines. G) Tumor weights of xenografts from the indicated cells (KYSE150 background). The data are the means ± s.e.m.; *n*  =  7. Two‐tailed *t* tests, ***p < 0.001, ns: not significant. H) Tumor weights of xenografts from the indicated cells (KYSE150 background) and treated with DDP, Z‐VAD, or S63845. The data are the means ± s.e.m.; *n*  =  7. Two‐tailed *t* tests, ***p < 0.001, **p < 0.01, ns: not significant. I) A schematic model showing the overlapping and distinct downstream events of OTUD1 overexpression and loss. OTUD1 overexpression not only activates both parthanatos and caspase‐dependent apoptosis pathways, but also compromises OXPHOS to further promotes cell death. In contrast, OTUD1 loss inhibits DDP‐induced apoptosis by suppression of parthanatos and caspase‐dependent apoptotic pathway.

We next asked what is the specific mechanism underlying OTUD1 recruitment of the CUL4A‐DDB1 complex. We first performed an in vitro co‐IP assay using V5‐OTUD1 with GST‐DDB1, GST‐CUL4A, or GST‐DCAF10. The results showed that only DCAF10 associated with OTUD1 in vitro (Figure S7A–C, Supporting Information). We asked whether OTUD1 modulated DCAF10 stability. CHX pulse‐chase assays showed that the overexpression of wild‐type OTUD1 significantly promoted DCAF10 stability (**Figure** **7**A; Figure S7D, Supporting Information). However, although OTUD1 depletion decreased DCAF10 protein level without CHX treatment, a significant decrease in the stability of DCAF10 proteins after OTUD1 depletion could not be confirmed by the CHX pulse‐chase assay because endogenous OTUD1 degraded more rapidly than DCAF10 (Figure 7B; Figure S7E, Supporting Information). Consistent with our previous results, neither OTUD1 overexpression nor depletion affected AIF stability (Figure S7D,E, Supporting Information). There was no sign of the degradation of AIF proteins at the longest timepoint of CHX treatment in our experimental set (20 h) (Figure S7D,E, Supporting Information), which indicates that AIF is a very stable protein.

Both in vivo and in vitro ubiquitination assays showed that OTUD1 deubiquitinates DCAF10 as a bona fide deubiquitinase (Figure S7F,G, Supporting Information). Co‐IP assays using the truncated mutants of DCAF10 and OTUD1 showed that the C terminus of DCAF10 is responsible for its interaction with OTUD1, while the Ala‐rich domain of OTUD1 interacts with DCAF10 (Figure S3A, S7H–J, Supporting Information). To confirm that DCAF10 is the key undertaker of the indirect modulation of OTUD1 towards MCL1, we depleted DCAF10 in KYSE150 cells. Ubiquitination assays showed that depletion of DCAF10 reduced the ubiquitination level of MCL1 and increased the MCL1 protein level (Figure 7C). Moreover, knockdown of either DCAF10 or CUL4A‐DDB1 in OTUD1‐overexpressing KYSE30 cells diminished the effect of OTUD1 on MCL1 ubiquitination (Figure 7D). IB for protein expression in ESCC cell lines indicated a positive correlation between the expression of OTUD1 and DCAF10 and a negative correlation between the expression of OTUD1 and MCL1, but no correlation of OTUD1 and AIF (Figure 7E), which further support our proposed regulatory model. To specify the type of deubiquitination event of OTUD1 on DCAF10, we repeated the deubiquitination assay with wild‐type, K48, and K63 ubiquitin mutant vectors. Unlike the effect of OTUD1 on AIF, OTUD1 primarily cleaved K48‐linked ubiquitin chains on DCAF10, which is the typical ubiquitin linkage type that contributes to protein degradation (Figure 7F).

We next asked whether the OTUD1‐DCAF10‐MCL1 axis functions in a way related to the effects of OTUD1 on AIF. To explain this, we depleted DCAF10 in KYSE150 cells with endogenous AIF mutations and performed xenografting experiments. As shown in Figure 7G and Figure S8A (Supporting Information), the depletion of DCAF10 showed no effect on the tumor suppressive role of the K244R mutant (comparing K244R&shDCAF10 to K244R), which is consistent with our proposed model that the K244R mutant of AIF triggers apoptosis via compromising OXPHOS. However, the depletion of DCAF10 rescued the tumor suppression of ectopic OTUD1 expression in K244R mutant cells (comparing K244R&D1&shDCAF10 to K244R&D1), which is due to the inhibition of the MCL1 downregulation‐induced caspase‐dependent apoptosis pathway and AIF release (parthanatos). Next, to specify which part of the proapoptotic functions of OTUD1 contributes to the increased chemoresistance of ESCC cells after OTUD1 depletion, we xenografted parental, K244R, and K255R KYSE150 cells with or without *OTUD1* knockout in nude mice then administered DDP alone or together with Z‐VAD or S63845 (an MCL1 inhibitor^[^ ^37^ ^]^). The partial rescue effects of Z‐VAD administration on chemoresistance were similar in the xenografts from the parental and K244R KYSE150 backgrounds, but Z‐VAD administration completely rescued DDP‐induced suppression of xenografts from the K255R KYSE150 background, indicating that the partial rescue effects of Z‐VAD on the xenografts from the parental and K244R KYSE150 backgrounds were due to the existence of AIF‐triggered caspase‐independent apoptosis (Figure 7H; Figure S8B, Supporting Information). The administration of S63845 rescued the chemoresistance caused by *OTUD1* depletion in all three backgrounds, which validated the critical role of MCL1 in the chemoresistance caused by *OTUD1* loss (Figure 7H; Figure S8B, Supporting Information). Theoretically, S63845 should lead to more effective suppression of DDP‐treated xenografts because the administration of S63845 should not only rescue the elevated MCL1 level triggered by *OTUD1* loss but also lead to extensive suppression of MCL1. Our results showed that S63845 only rescued the chemoresistance triggered by OTUD1 depletion in parental and K244R backgrounds, but it caused excessive suppression of xenografts in K255R background (Figure 7H; Figure S8B, Supporting Information). These results may be explained that although the deubiquitination effect of OTUD1 on AIF K244 triggered cell death by compromised OXPHOS, the sustained OXPHOS by *OTUD1* depletion cannot antagonize the apoptosis pathways triggered by DDP. For the same reason, DDP only triggers caspase‐dependent apoptosis pathway in the K255R background, and thus S63845 treatment leads to an excessive suppression effect on the chemoresistance caused by OTUD1 loss (Figure 7I).

## 3 Conclusion and Discussion

ESCC contributes to a significant proportion of morbidity and mortality in developing countries, and most cases were diagnosed in Asia and Sub‐Saharan Africa.^[^ ^1^ ^]^ The incidence of ESCC has broadly decreased in recent years, probably due to improved economic gains and dietary improvements in high‐risk areas. However, ESCC patients still suffer a poor prognosis with limited therapeutic options to control chemoresistance and metastasis.^[^ ^38^ ^]^ OTUD1 has recently been recognized as a tumor suppressor that suppresses the growth and metastasis of breast cancer,^[^ ^39^, ^40^ ^]^ but its role in neither ESCC nor chemoresistance remains unknown. Through an in vivo screening model, we found that ESCC cells with *OTUD1* loss exhibits resistance to DDP treatment. Subsequent studies confirmed the essential role of OTUD1 in promoting chemosensitivity via apoptosis induction. Interestingly, we showed that OTUD1 only promotes the apoptosis of ESCC cells, but it does not affect their proliferation rate. Moreover, although high OTUD1 expression significantly correlated with a favorable patient prognosis in ESCC, it does not correlate with other clinical variables, such as pathological stage or metastasis. These results indicate that the role of OTUD1 in ESCC is restricted to its proapoptotic function, but it is sufficient to affect the overall survival of ESCC patients.

Escape from apoptosis is a common cause of cancer cell resistance to chemotherapies. Most studies of chemoresistance focused on caspase‐dependent apoptotic signaling.^[^ ^41^, ^42^, ^43^ ^]^ However, the caspase‐independent apoptotic pathway also plays an important, yet poorly defined, role in apoptotic events. AIF maintains mitochondrial structure and OXPHOS under normal conditions and induces parthanatos after nuclear translocation in response to apoptotic stimuli. Because the two functions of AIF occur in different intracellular locations, scientists have engaged in studies aiming to separate the two functions of AIF.^[^ ^21^, ^22^ ^]^ However, no factor that can conjunctively control the two functions of AIF in mitochondria and nuclei was revealed. The present study identified OTUD1 as a modulator of AIF that links the two distinct roles of AIF. In mitochondria, OTUD1‐mediated AIF deubiquitination disrupts the mitochondrial structure and compromises OXPHOS, which results in a lack of ATP production and subsequent cell death. In the nuclei, OTUD1‐deubiquitinated AIF gains an increased DNA‐binding ability and promotes parthanatos. We showed that both K244 and K255 in AIF are essential for OTUD1‐mediated AIF deubiquitination. Specifically, K244 is critical for maintaining the mitochondrial structure and OXPHOS, while K255 endows AIF with its DNA‐binding ability.

Although calpain was previously verified to cleave AIF and this process has been suggested to be critical for AIF release from mitochondria,^[^ ^44^, ^45^ ^]^ subsequent studies argued that truncation is not required for AIF nuclear translocation during parthanatos.^[^ ^46^, ^47^ ^]^ Notably, our study is consistent with the model that no truncation is needed before the release of AIF. One reasonable explanation may be that whether the release of AIF needs truncation depends on the specific cell types, and more importantly, the inducing factors for its release. CHIP and USP2 were previously identified as an E3 ligase and a deubiquitinase that control the protein stability of truncated AIF (tAIF).^[^ ^48^ ^]^ However, the study of CHIP and USP2 was performed on a research background that AIF must be cleaved into tAIF before its release from mitochondria. Despite this difference, these two studies share a coincident finding that the ubiquitin‐proteasome system could not degrade AIF, at least in mitochondria. Another E3 ligase, XIAP, promotes nondegradative ubiquitination of AIF and affects its DNA binding ability,^[^ ^23^ ^]^ but no deubiquitinase was found to mediate the deubiquitination of AIF. Our study for the first time showed that OTUD1 functions as a bona fide deubiquitinase of AIF. OTUD1 is located in the mitochondrial intermembrane space and the cytoplasm, and the deubiquitination of AIF by OTUD1 is not related to protein stability.

MCL1 is an antiapoptotic member of the BCL‐2 family, and recent studies have revealed its essential role in chemoresistance in cancer.^[^ ^49^, ^50^, ^51^ ^]^ The BCL‐2 family controls the caspase‐dependent apoptotic pathway primarily by affecting the release of cytochrome C in mitochondria.^[^ ^52^, ^53^ ^]^ However, increased mitochondrial membrane permeability also leads to the release of AIF into the cytoplasm and induces the caspase‐independent apoptotic pathway.^[^ ^33^, ^54^ ^]^ The present study revealed that OTUD1 stabilizes DCAF10 to indirectly promote the ubiquitination and degradation of MCL1 by recruiting the CUL4A‐DDB1 ubiquitin ligase. The decreased protein level of MCL1 acts as an inducing factor for AIF release. In the context of *AIF* knockout, OTUD1 still exerted pro‐apoptotic effects via the caspase‐dependent apoptotic pathway. MCL1 inhibitors, like S63845, are widely used in clinical trials and show great potential for application in the treatment of chemoresistant cancers. The OTUD1‐induced activation of caspase‐dependent apoptosis and parthanatos begins with MCL1 degradation. Therefore, we hypothesized that targeting MCL1 would rescue the chemoresistance triggered by OTUD1 loss. Indeed, S63845 rescued the resistance to DDP treatment of OTUD1‐depleted xenografts, and these results support MCL1 inhibitors as an effective means of selectively targeting chemoresistant cancer cells with low OTUD1 expression. It's worth noting that the MCL1 targeting strategy only rescued the OTUD1 loss‐induced chemoresistance that is caused by the inactivation of caspase‐dependent apoptosis pathway and parthanatos. However, our results demonstrated that ectopic expression of OTUD1 promotes ESCC cell death by inducing the caspase‐dependent apoptotic signaling, parthanatos, and compromising OXPHOS. Consequently, specifically rescuing or overexpressing OTUD1 in cancer cells may be a better strategy than only interfering with MCL1 (Figure 7I).

Interestingly, although OTUD1 acts as a bona fide DUB for both AIF and DCAF10, its modes of action on these two substrates are completely different. OTUD1 cleaves the K27‐ and K63‐linked ubiquitin chains on AIF via an enzymatic‐dependent manner to modulate AIF activity without stabilization, but the association between OTUD1 and AIF is not interrupted by inactive mutants of OTUD1. In contrast, OTUD1 cleaves K48‐linked ubiquitin chains on DCAF10 to stabilize the DCAF10 protein, and the association between OTUD1 and DCAF10 relies on the enzymatic activity of OTUD1. These results indicate a subtle modulatory mode in which one DUB acts on two substrates via different mechanisms but eventually contributes to a harmonized biological effect. In summary, our study reveals a central role of OTUD1 in both caspase‐independent and caspase‐dependent apoptotic pathways, and the mechanism underlying the proapoptotic role of OTUD1 can be divided into three steps: 1, AIF deubiquitination disrupts the mitochondrial structure and compromises OXPHOS to block energy support and induce cell death; 2, ubiquitination‐mediated MCL1 degradation increases the permeability of the mitochondrial membrane and promotes the release of cytochrome C and AIF; and 3, the released cytochrome C activates caspase‐dependent apoptosis, and deubiquitinated AIF acquires an increased DNA‐binding ability to promote parthanatos (Figure S8C, Supporting Information).

## 4 Experimental Section

##### Cell Culture and Plasmids

The human ESCC cell lines were kindly provided by Dr. Yutaka Shimada ^[^ ^55^ ^]^ and cultured in RPMI‐1640 medium supplemented with 10% FBS. The 293T cell line was purchased from the American Type Culture Collection (ATCC, VA, USA) and cultured in DMEM supplemented with 10% FBS. All cells were maintained in a humidified cell incubator with 5% CO_2_ at 37 °C and were routinely authenticated using short tandem repeat (STR) DNA fingerprinting. All cells were tested using the MycoBlue Mycoplasma Detector (Vazyme Biotech, Nanjing, China) to exclude Mycoplasma contamination before use. The chemoresistant cell lines YES2/DDP and YES2/PTX were established from a parental YES2 cell line. In brief, parental cells were exposed to high doses of DDP or PTX for 2 h then cultured in complete medium. When cell growth was in the logarithmic phase, cells were re‐exposed to high doses of the drugs. After 6–10 cycles, the chemoresistant cell lines were established, and the chemoresistance indexes were calculated. Full‐length or truncated cDNAs were generated using a PCR‐based method with specific primers and subsequently cloned into the pLVX‐IRES‐neo or pcDNA3 vector. The mutant plasmids were constructed using a Q5 Site‐Directed Mutagenesis Kit (New England Biolabs, MA, USA) following the manufacturer's instructions.

##### Antibodies and Reagents

The antibodies used in this study were used at the dilutions listed: anti‐OTUD1, 1:1000 (IB; Abcam, MA, USA, ab122481), 1:100 (IHC; Abcam, ab122481), 1:150 (IP; Abcam, ab122481), and 1:50 (PLA; Abcam, ab122481); anti‐BAP1, 1:1000 (IB; Cell Signaling Technology, MA, USA, #13 271); anti‐MCL1, 1:1000 (IB; Cell Signaling Technology, #94 296) and 1:100 (IP; Cell Signaling Technology, #94 296); anti‐AIF, 1:1000 (IB; Cell Signaling Technology, #4642); anti‐AIF, 1:20 (IP; Cell Signaling Technology, #5939); anti‐AIF, 1:50 (PLA; Abcam, ab110327); anti‐IgG, 1:20 (IP; Cell Signaling Technology, #3423); anti‐V5‐Tag, 1:1000 (IB; Cell Signaling Technology, #13 202); anti‐Flag‐Tag, 1:1000 (IB; Cell Signaling Technology, #8146); anti‐Myc‐Tag, 1:1000 (IB; Cell Signaling Technology, #2276); anti‐HA‐Tag, 1:5000 (IB; Abcam, ab9110); anti‐GST‐Tag, 1:1000 (IB; Cell Signaling Technology, #2622); anti‐His‐Tag, 1:1000 (IB; Cell Signaling Technology, #12 698); anti‐Ubiquitin, 1:1000 (IB; Cell Signaling Technology, #3936); anti‐Cleaved caspase‐3, 1:1000 (IB; Cell Signaling Technology, #9664); anti‐Caspase‐9, 1:1000 (IB; Cell Signaling Technology, #9502); anti‐PARP, 1:1000 (IB; Cell Signaling Technology, #9542); anti‐BCL‐2, 1:1000 (IB; Cell Signaling Technology, #4223); anti‐BCL‐W, 1:1000 (IB; Cell Signaling Technology, #2724); anti‐BCL‐XL, 1:1000 (IB; Cell Signaling Technology, #2764); anti‐OXPHOS antibody cocktail, 1:500 (IB; Abcam, ab110411); anti‐DCAF10, 1:1000 (IB; Abcam, ab179400); anti‐CUL4A, 1:1000 (IB; Abcam, ab92554); anti‐DDB1, 1:1000 (IB; Abcam, ab109027); anti‐MIF, 1:1000 (IB; Abcam, ab175189); anti‐TOMM20, 1:1000 (IB; Abcam, ab56783) and 1:50 (PLA; Abcam, ab56783); anti‐CHCHD4, 1:500 (IB; Santa Cruz Biotechnology, TX, USA, sc‐365137); anti‐Smac, 1:1000 (IB; Cell Signaling Technology, #2954) and 1:50 (PLA; Cell Signaling Technology, #2954); anti‐PAR, 1:500 (IB; Abcam, ab14459); anti‐Histone H3, 1:10 000 (IB; Abcam, ab1791); anti‐GAPDH, 1:5000 (IB; Sigma‐Aldrich, MO, USA, G9545); anti‐*β*‐actin, 1:4000 (IB; Sigma‐Aldrich, A5316); and normal rabbit IgG (IP; Cell Signaling Technology, #2729). Cycloheximide (CHX, HY‐12320), Z‐VAD‐FMK (Z‐VAD, HY‐16658B), and S63845 (HY‐100741) were obtained from MedChemExpress (MCE, NJ, USA). MG132 (T2154) was purchased from TargetMol (MA, USA). JC‐1 MitoMP Detection Kit (MT09) was purchased from Dojindo Laboratories (Kumamoto, Japan).

##### Quantitative Real‐Time PCR (qRT‐PCR)

Total RNA extraction from cultured cells was performed using TRIzol reagent (Thermo Scientific, MA, USA), and RNA was subsequently reverse transcribed to cDNA using a Quantscript RT Kit (Tiangen, Beijing, China). qRT‐PCR analysis was performed in a StepOnePlus Real‐Time PCR system (Applied Biosystems, CA, USA). The relative expression levels of the target genes were normalized to those of the housekeeping gene GAPDH. The OTUD1 (HQP058247)‐ and DUB3 (HQP011104)‐specific primers used in the qRT‐PCR analyses were purchased from FulenGen (Guangzhou, China). The remaining qRT‐PCR primers are shown in Table S1, Supporting Information.

##### Virus Production and Cell Infection

Lentivirus was produced using 293T cells with the second‐generation packaging system psPAX2 (#12 260, Addgene, MA, USA) and pMD2.G (#12 259, Addgene). Transfections were performed using Hieff Trans Liposomal Transfection Reagent (40 802, Yeasen, Shanghai, China). The shRNA sequences targeting OTUD1 were as follows: sh1, GCAGATGCTGAATGTGAATAT and sh2, GCGACGAAGAACTTGCCAAAT. The shRNA sequences targeting DCAF10 were as follows: sh1, GGTGAAGAACATCGAATATGA and sh2, GGGTTACATCAAAGAACTTTG. The shRNA sequences targeting CUL4A were as follows: sh1, GCAGAACTGATCGCAAAGCAT and sh2, GGACAAGAAGATGTTACTAAA. The shRNA sequences targeting DDB1 were as follows: sh1, CGACTCAATAAAGTCATCAAA and sh2, CGTGTACTCTATGGTGGAATT. The following nontargeting shRNA sequence was used as the negative control: AGTCTTAATCGCGTATAAGGC. The sgRNA sequences were designed using an online CRISPR design tool (http://tools.genome-engineering.org).^[^ ^56^ ^]^

##### CRISPR Knock‐In

Guide‐it Complete sgRNA Screening Systems (632 636; Clontech, CA, USA) were used for the in vitro transcription and efficiency testing of sgRNAs. Donor DNAs with mutant sites were produced using the Guide‐it Long ssDNA Production System (632 644; Clontech). sgRNAs, donor DNAs, together with Guide‐it Recombinant Cas9 (632 640; Clontech) were imported into cells via electroporation using the Neon Transfection System (MPK5000; Invitrogen, CA, USA). Cells were allowed to recover for 72 h after electroporation then seeded into 96‐well plates for single clone selection. After primary selection by the Guide‐it Knockin Screening Kit (632 660; Clontech), positive clones were further validated by DNA sequencing for successful mutation of targeted sites.

##### Immunoprecipitation and Western Blotting

Cell lysates were prepared using RIPA lysis buffer (CW2333; CoWin Biosciences, Jiangsu, China) with freshly added protease inhibitor cocktail (0 469 315 9001; Roche, Basel, Switzerland) for 20 min on ice. For co‐IP and ubiquitination assays, Cell Lysis Buffer for Western and IP (P0013; Beyotime Biotechnology, Shanghai, China) were used. Nuclear proteins were separated into soluble parts and chromatin‐associated parts using the Detergent‐free nuclear Matrix Protein Isolation Kit (NM‐033; Invent Biotechnologies, MN, USA). Intact mitochondria were isolated using the Minute Mitochondrial Isolation Kit for Mammalian Cells and Tissues (MP‐007; Invent Biotechnologies). The protein concentrations were measured using a BCA assay kit (Thermo Scientific). For immunoprecipitation, equal amounts of lysate were incubated overnight at 4 °C with anti‐Flag magnetic beads (M8823, Sigma‐Aldrich), anti‐Myc magnetic beads (HY‐K0206, MCE), anti‐V5 magnetic beads (M167‐11, Medical & Biological Laboratories, Nagoya, Japan) or protein A/G magnetic beads (HY‐K0202, MCE) and an anti‐MCL1/AIF/OTUD1 antibody. The beads were washed three times with cell lysis buffer, and the immunoprecipitated protein complexes were analyzed using IB with the indicated antibodies. The proteins were resolved on 8–15% SDS‐PAGE gels depending on the molecular weight and transferred onto PVDF membranes (Merck Millipore, MA, USA).

##### MS to Identify OTUD1‐Associated Proteins

MS was performed as previously described.^[^ ^57^ ^]^ Briefly, Flag‐OTUD1 was transfected into 293T cells, and cell lysates were immunoprecipitated with anti‐Flag magnetic beads. The beads were washed three times with cell lysis buffer and eluted with 1 × loading buffer. The eluted proteins were separated on 10% gels, silver stained with Pierce Silver Stain (24 600, Thermo Scientific) and subjected to gel‐based liquid chromatography‐tandem mass spectrometry (LC‐MS/MS) and data analysis using MaxQuant software (version 1.5.3.30) against the UniProtKB/Swiss‐Prot human database.

##### MS to Identify Ubiquitination Sites on AIF

Flag‐AIF was transfected into 293T cells. Forty‐eight hours later, cell lysates were collected and immunoprecipitated with anti‐Flag magnetic beads. The beads were washed three times with cell lysis buffer and eluted with 1× loading buffer. The eluate was collected and resolved using 10% SDS‐PAGE. Gels were silver stained using Pierce Silver Stain for Mass Spectrometry according to the manufacturer's instructions. The band containing accumulated Flag‐AIF was excised and subjected to gel‐based LC‐MS/MS and data analysis. The ubiquitination sites were identified using the MASCOT search engine (Matrix Science, London, UK; version 2.2) embedded into Proteome Discoverer 1.4 (Thermo Electron, CA, USA).

##### Recombinant Protein Purification

cDNA sequences were cloned into the pGEX‐6P‐1 vector containing an N‐terminal GST tag protein, expressed in BL21(DE3) cells and cultured in LB medium containing ampicillin at 37 °C. Isopropyl‐*β*‐D‐galactosidase (0.5 × 10^−3^ m) was added at an OD600 of 1, and recombinant protein expression was induced at 16 °C overnight. Recombinant GST‐tagged protein was purified with a Pierce GST Spin Purification Kit (16 107, Thermo Scientific) following the manufacturer's instructions.

##### Ubiquitination Assay

The ubiquitination assay was performed as previously described.^[^ ^51^, ^57^ ^]^

##### Immunofluorescence Staining

Cells were seeded in a µ‐Slide V1 (ibidi, Martinsried, Germany), and immunofluorescence experiments were performed as previously described.^[^ ^57^ ^]^ PLA assays were performed using Duolink In Situ Red Starter Kit (DUO92101, Sigma‐Aldrich) following the manufacturer's instructions.

##### Flow Cytometry

Flow cytometry was performed using an apoptosis detection kit (Dojindo Laboratories) according to the manufacturer's protocol, as previously described.^[^ ^58^ ^]^

##### Cell Counting Kit‐8 (CCK‐8) Cell Proliferation and Cell Viability Assays

CCK‐8 reagent (Dojindo Laboratories) was added to the cell culture medium at a ratio of 1:10, and the absorbance was measured at 450 nm after incubation for 1 h at 37 °C. For the cell proliferation assay, 2.5 × 10^3^ cells were seeded in 96‐well microplates, and one plate was measured each day for five consecutive days. The relative cell viability (fold) was calculated by normalizing the absorbance at 450 nm on days 2–5 to the absorbance measured on day 1. For the cell viability assay, 1 × 10^4^ ESCC cells were seeded in 96‐well microplates, and drugs were added after cell adhesion. Viability was measured 24 h later. Cell viability (%) was calculated by normalizing the absorbance at 450 nm of the experimental groups to that of the control group.

##### Animal Experiments

All animal protocols were approved by the Animal Care and Use Committee of the Chinese Academy of Medical Sciences Cancer Hospital. For subcutaneous xenografting, 1 × 10^6^ ESCC cells were subcutaneously implanted into 6‐week‐old male BALB/c nude mice (HFK Bioscience, Beijing, China). Mice were fed a normal diet and administered saline or DDP (6 mg kg^−1^) three times weekly until sacrifice. Z‐VAD (2 mg kg^−1^) and S63845 (25 mg kg^−1^) were administered for 5 continuous days 1 week after xenografting. Tumor volumes were calculated using the following formula: 0.52 × length × width^2^. After the tumors had grown for the designated time, all mice were euthanized, and the tumors were harvested.

##### Seahorse Assays

The OCR and ECAR were measured with a Seahorse XF96 Extracellular Flux Analyzer (Agilent Technologies, CA, USA) using a Cell Mito Stress Test Kit (103 015, Agilent Technologies) and a Glycolysis Stress Test Kit (103 020, Agilent Technologies), respectively. Briefly, 5×10^3^ cells were plated into Seahorse XF96 Cell Culture Microplates (101 085, Agilent Technologies). The culture medium was replaced with XF RPMI Base Medium (103 575, Agilent Technologies) supplemented with 1 ×10^−3^ m pyruvate, 2 ×10^−3^ m glutamine, and 10 ×10^−3^ m glucose (for OCR experiments) or 1 ×10^−3^ m glutamine (for ECAR experiments) and incubated in a non‐CO_2_ incubator for 1 h before the experiments were performed. For the mitochondrial stress test, oligomycin, carbonyl cyanide 4‐ (trifluoromethoxy) phenylhydrazone (FCCP) and rotenone/antimycin A (Rot/AA) were sequentially injected to final concentrations of 1.5 × 10^−6^ m, 0.5 (KYSE30) or 1.0 (KYSE150) × 10^−6^ m, and 0.5 × 10^−6^ m, respectively. For the glycolysis test, glucose, oligomycin and 2‐deoxy‐D‐glucose (2‐DG) were sequentially injected to final concentrations of 10 × 10^−3^, 1 × 10^−6^ , and 50 × 10^−3^ m, respectively. Data analyses were performed using Wave software (Agilent Technologies) with five replicates per group.

##### ChIP Assay

ChIP assays were performed using a ChIP kit (#9003, Cell Signaling Technology) according to the manufacturer's protocol. The primer sequences for qPCR detection were as follows (5’‐3’): *GAPDH*‐F, TCTGCTTCTCTGCTGTAGGCTCAT; *GAPDH*‐R, GAGGCTGTTGTCATACTTCTCATGGT; *RPL30*‐F, AGAGGTTGCAGTGAGCTTAGATTGTG, *RPL30*‐R, TCTGATTTCATTGGATCTGGGATGGG.

##### Patient Samples and IHC

Paraffin‐embedded in situ tumor tissue blocks were collected from 122 patients with ESCC during their initial treatment at Zhejiang Cancer Hospital. This study was approved by the Institutional Research Ethics Committee. Written informed consent was obtained from all patients. Tissue sections were placed in a 60 ℃ incubator overnight, dewaxed in xylene and rehydrated in gradient ethanol. The slides were treated with 3% hydrogen peroxide for 30 min in a moist chamber, followed by antigen retrieval using 5 × 10^−3^ m citrate buffer (pH 6.0) for 30 min. After blocking with 5% BSA, slides were incubated overnight with relevant primary antibodies at 4 ℃, then incubated with the corresponding secondary antibodies for 1 h at room temperature. The sections were incubated with diaminobenzidine and counterstained with hematoxylin. IHC staining results were converted to H‐scores as follows: H‐score = ∑pi×i, where pi represents the percentage of positive cells (0–100) and i represents the staining intensity (0: negative, 1: weak, 2: medium, and 3: strong). IHC staining was scored by two independent observers.

##### Statistical Analysis

Two‐tailed Student's *t* test, two‐way ANOVA test, or Kaplan‐Meier test were used for statistical analyses. For all statistical analyses, differences for which *p* ≤ 0.05 were considered statistically significant, and at least three biologically independent experiments with similar results were reported. The data are presented as the means ± s.e.m. or means ± s.d. as indicated in the figure legends. The sample size (*n*) for each statistical analysis was determined on the basis of pretests and previous similar experiments, and are indicated detailly in the figure legends. Data analyses were performed using GraphPad Prism (version 8.01, GraphPad Software, CA, USA).

## Conflict of Interest

The authors declare no conflict of interest.

## Supporting information

Supporting Information
