# Supplementary material for: OTUD1 Activates Caspase‐Independent and Caspase‐Dependent Apoptosis by Promoting AIF Nuclear Translocation and MCL1 Degradation
Source: Adv Sci (Weinh). 2021 Feb 8;8(8):2002874. doi: 10.1002/advs.202002874 (PMC8061361; doi:10.1002/advs.202002874)
Supplement: Supplementary file 1 — Supporting Information [file ADVS-8-2002874-s001.pdf]

© 2021 Wiley-VCH GmbH

ADVANCED SCIENCE
Open Access

Supporting Information

for *Adv. Sci.,* DOI: 10.1002/advs.202002874

OTUD1 Activates Caspase-Independent and Caspase-Dependent Apoptosis by Promoting AIF Nuclear Translocation and MCL1 Degradation

*Qingyu Luo, Xiaowei Wu, Pengfei Zhao, Yabing Nan, Wan Chang, Xiaolin Zhu, Dan Su, Zhihua Liu**

Supporting Information

OTUD1 Activates Caspase-Independent and Caspase-Dependent Apoptosis by Promoting AIF Nuclear Translocation and MCL1 Degradation

*Qingyu Luo, Xiaowei Wu, Pengfei Zhao, Yabing Nan, Wan Chang, Xiaolin Zhu, Dan Su, Zhihua Liu**

E-mail: liuzh@cicams.ac.cn

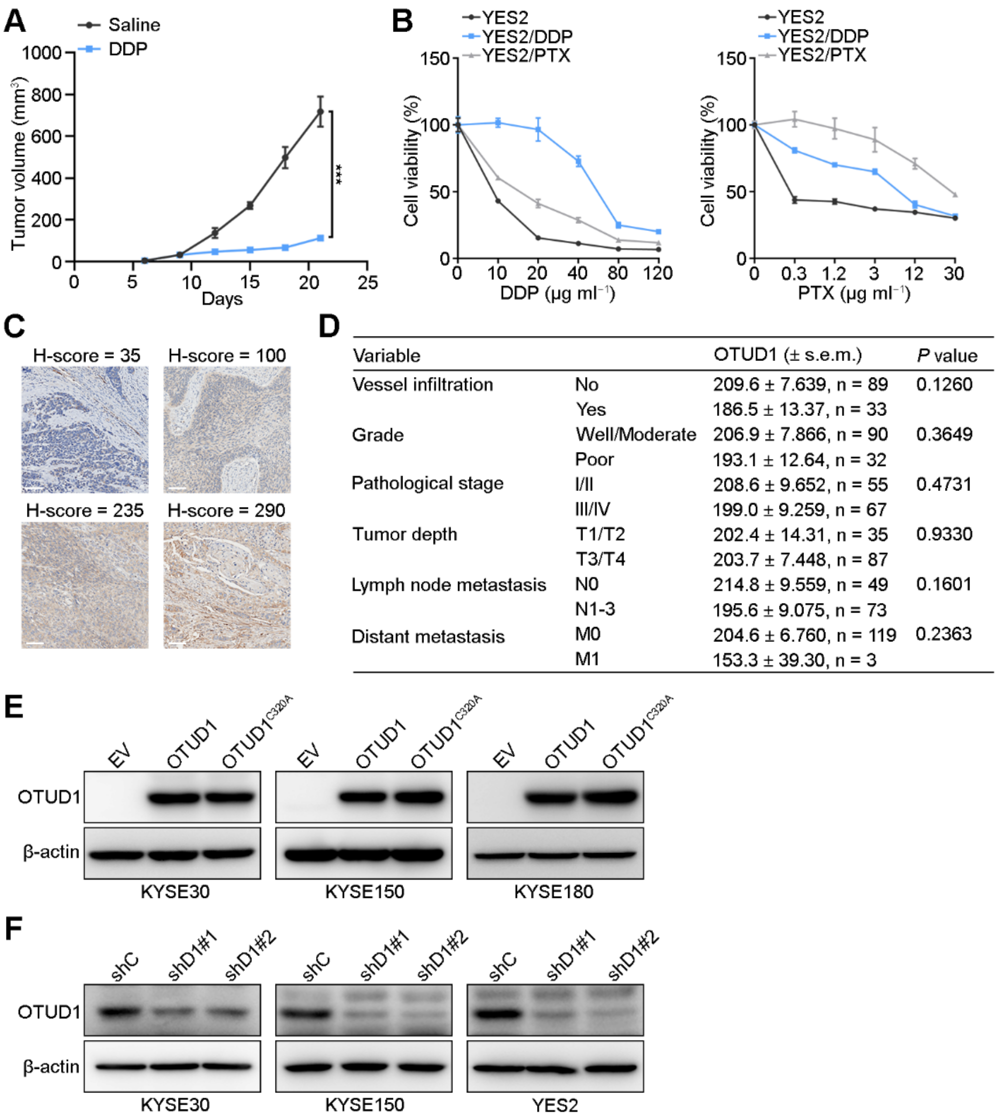

Variable		OTUD1 (± s.e.m.)	*P* value
Vessel infiltration	No	209.6 ± 7.639, n = 89	0.1260
	Yes	186.5 ± 13.37, n = 33	
Grade	Well/Moderate	206.9 ± 7.866, n = 90	0.3649
	Poor	193.1 ± 12.64, n = 32	
Pathological stage	I/II	208.6 ± 9.652, n = 55	0.4731
	III/IV	199.0 ± 9.259, n = 67	
Tumor depth	T1/T2	202.4 ± 14.31, n = 35	0.9330
	T3/T4	203.7 ± 7.448, n = 87	
Lymph node metastasis	N0	214.8 ± 9.559, n = 49	0.1601
	N1-3	195.6 ± 9.075, n = 73	
Distant metastasis	M0	204.6 ± 6.760, n = 119	0.2363
	M1	153.3 ± 39.30, n = 3	

Figure S1. OTUD1 correlates with chemosensitivity in ESCC. A) *In vivo* growth of xenografts in mice treated with saline or DDP (6 mg kg^{-1}). The data are the means ± s.e.m.; $n = 4$. Two-tailed *t* tests, $***P < 0.001$. B) Relative viability of YES2/DDP, YES2/PTX and YES2 cells treated with DDP or PTX. The data are the means ± s.d.; $n = 5$. C) Representative immunohistochemical staining images and H-scores of OTUD1. Scale bars, 100 μm. D) Correlations between OTUD1 expression and clinical variables in patients with ESCC. Two-tailed *t* tests. E,F) IB for OTUD1 expression in the indicated cells.

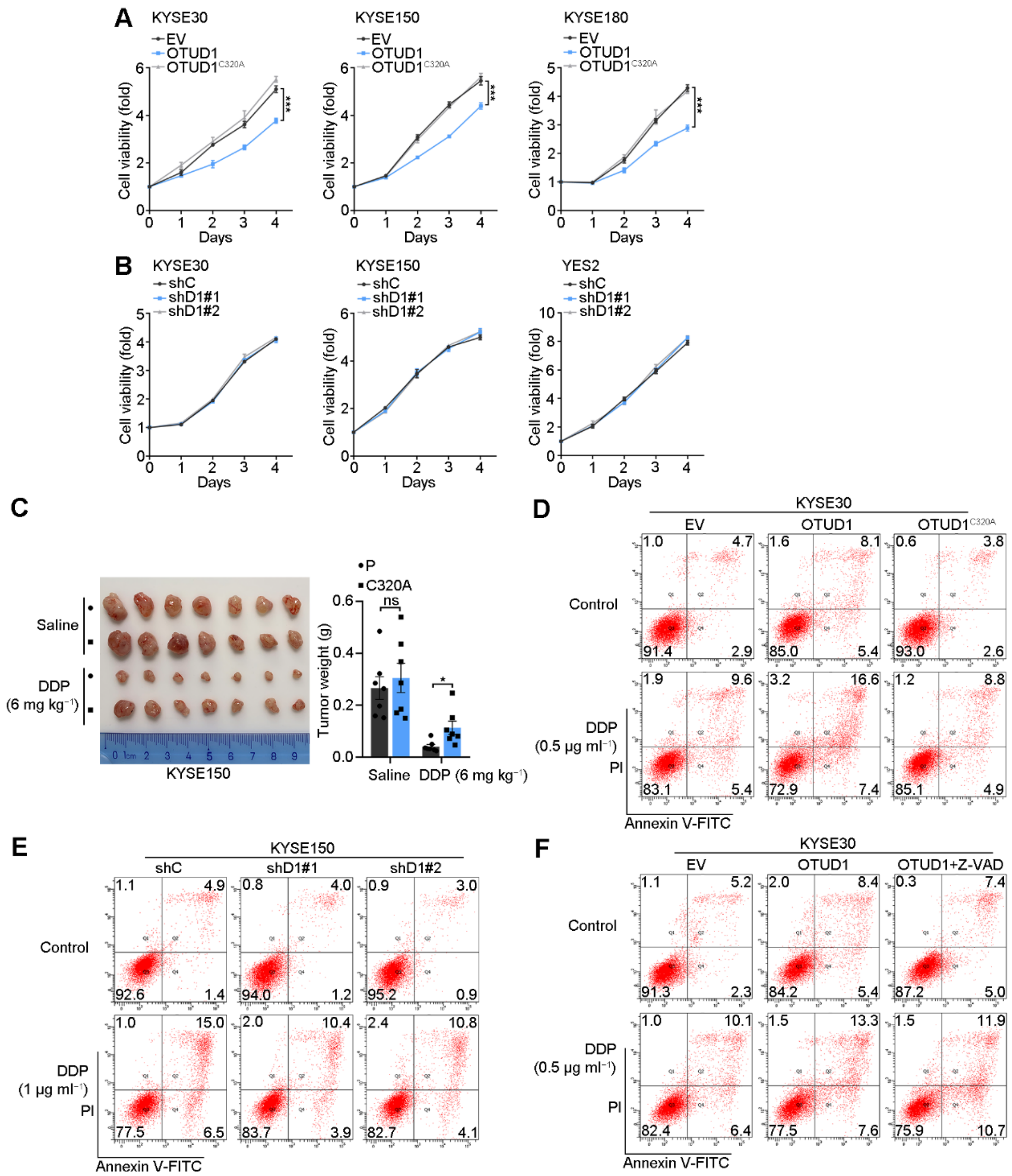

Figure S2. OTUD1 activates the caspase-independent apoptotic pathway. A) *In vitro* growth of ESCC cells expressing EV, OTUD1 or OTUD1^{C320A}. The data are the means ± s.d.; $n = 5$. Two-tailed t tests, $***P < 0.001$. B) *In vitro* growth of ESCC cells expressing shC, shD1#1, or shD1#2 shRNAs. The data are the means ± s.d.; $n = 5$. C) Representative image and tumor weights of xenografts from parental or endogenously mutated KYSE150 cells treated with saline or DDP. The data are the means ± s.e.m.; $n = 7$. Two-tailed t tests, $*P < 0.05$, ns: not significant. D-F) Representative images of the apoptotic fraction of the indicated cells as determined using flow cytometric analysis.

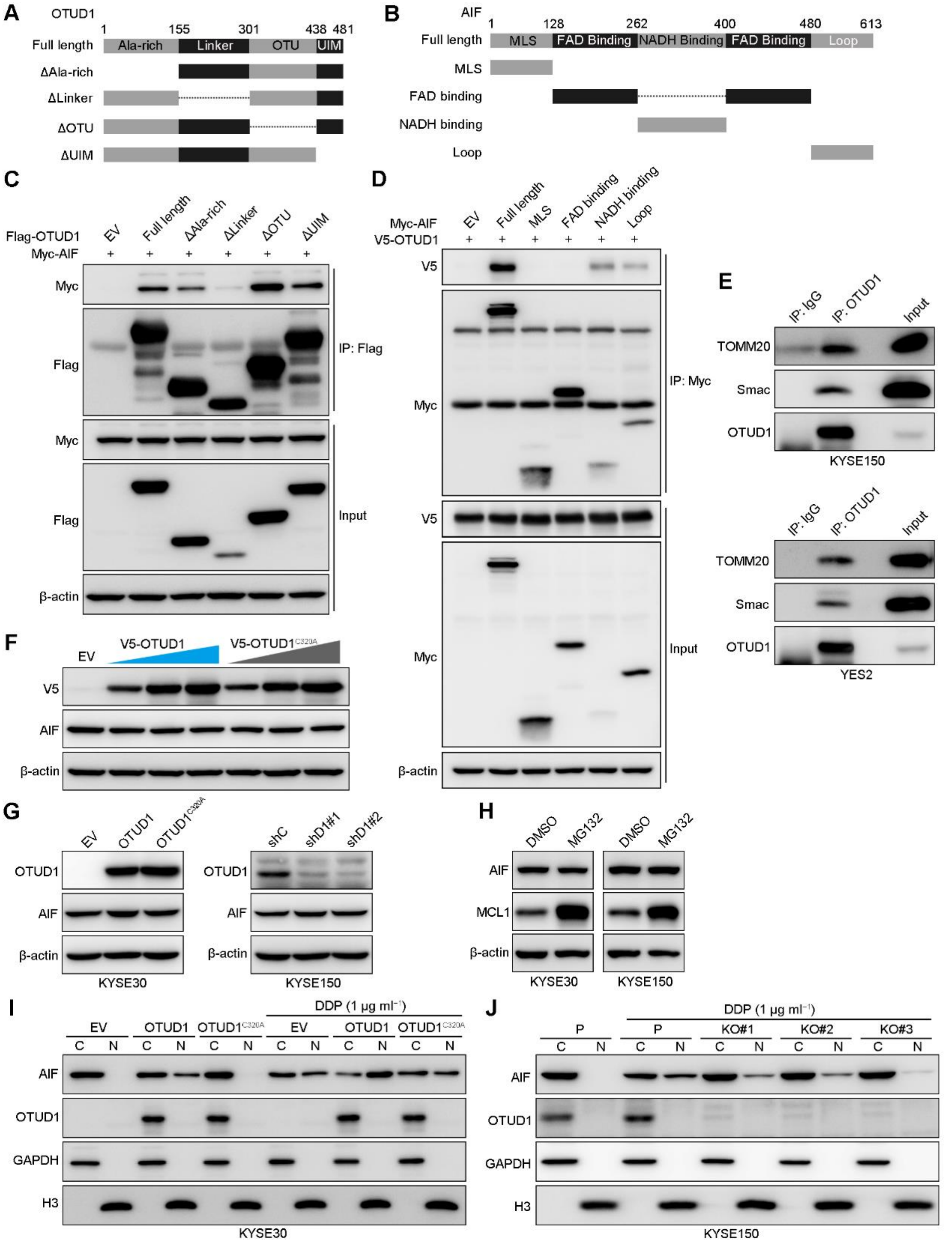

Figure S3. OTUD1 interacts with AIF. A,B) Schematics showing truncated mutants of OTUD1 and AIF. C,D) IB for the indicated proteins in co-IP assays. E) IB for the indicated proteins in co-IP assays using anti-OTUD1 antibody. F) IB for the indicated proteins in 293T cells transfected with increasing amounts of OTUD1 or OTUD1^{C320A}. G) IB for the indicated proteins in KYSE30 cells expressing EV, OTUD1 or OTUD1^{C320A} and in KYSE150 cells expressing shC, shD1#1, or shD1#2 shRNAs. H) IB for AIF and MCL1 expression in KYSE30 and KYSE150 cells treated with or without MG132 for 8 hours. I,J) IB for AIF distribution in the indicated cells (C: cytoplasm; N: nucleus).

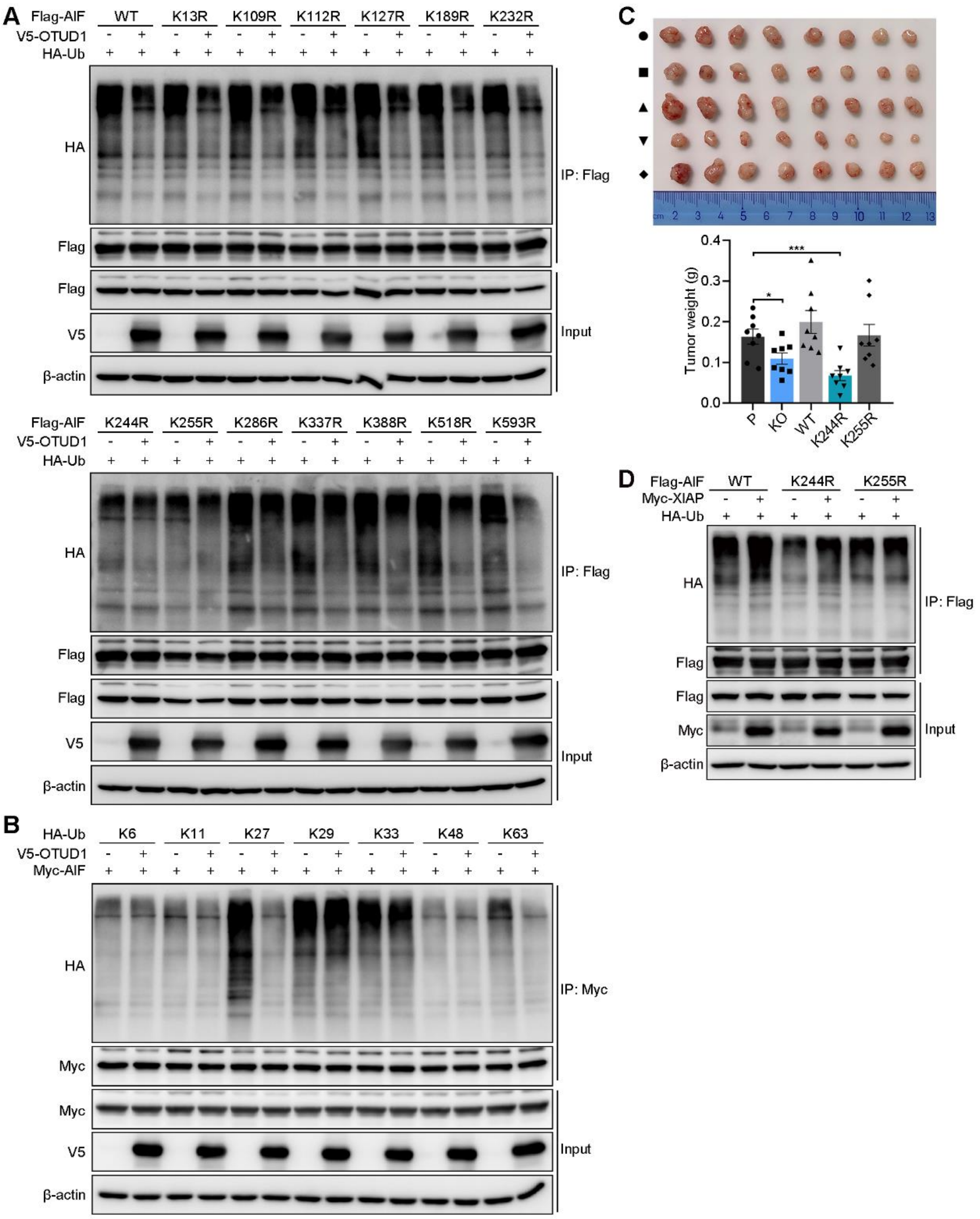

Figure S4. OTUD1 mediates the cleavage of K27- and K63-linked ubiquitin chains on K244 and K255 of AIF. A) IB to assess the ubiquitination level of AIF mutants in 293T cells cotransfected with Flag-AIF mutants, V5-OTUD1 and HA-Ub. B) IB for AIF ubiquitination in 293T cells cotransfected with Myc-AIF, V5-OTUD1 and the HA-Ub mutants. K6, K11, K27, K29, K33, K48 and K63 indicate that all lysines, except K6, K11, K27, K29, K33, K48 or K63, respectively, were mutated to arginines. C) Representative image and tumor weights of xenografts from the indicated cells (P: parental; KO: *AIF*-knockout; WT: AIF^{WT}; K244R: AIF^{K244R}; K255R: AIF^{K255R}). The data are the means ± s.e.m.; n = 8. Two-tailed t tests, *** P <0.001, *P < 0.05. D) IB for ubiquitination level of Flag-AIF mutants in 293T cells cotransfected with Flag-AIF mutants, Myc-XIAP and HA-Ub.

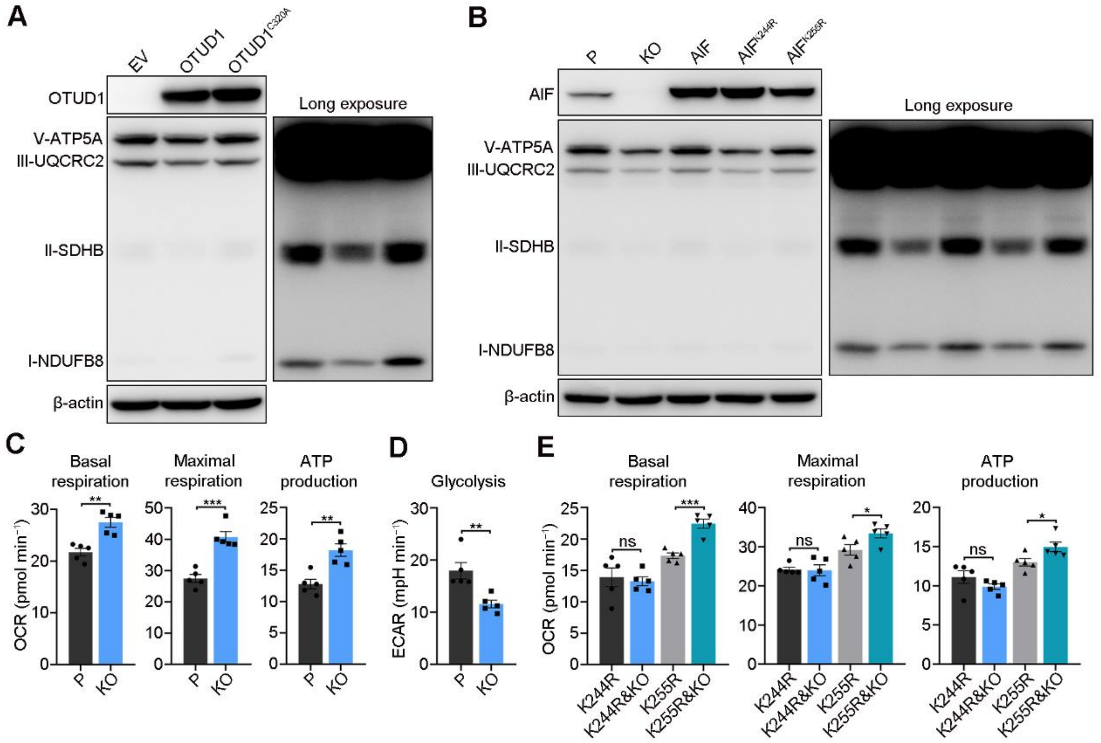

Figure S5. OTUD1 compromises OXPHOS via deubiquitination of AIF K244. A,B) IB for respiratory chain proteins in the indicated cells (KYSE30 background). C-E) Statistical analyses of basal respiration, maximal respiration, ATP production (C,E) and basal glycolysis rate (D) in the indicated cells (KYSE150 background). The data are the means ± s.e.m.; $n = 5$. Two-tailed t tests, $^{***}P < 0.001$, $^{**}P < 0.01$, $^{*}P < 0.05$, ns: not significant.

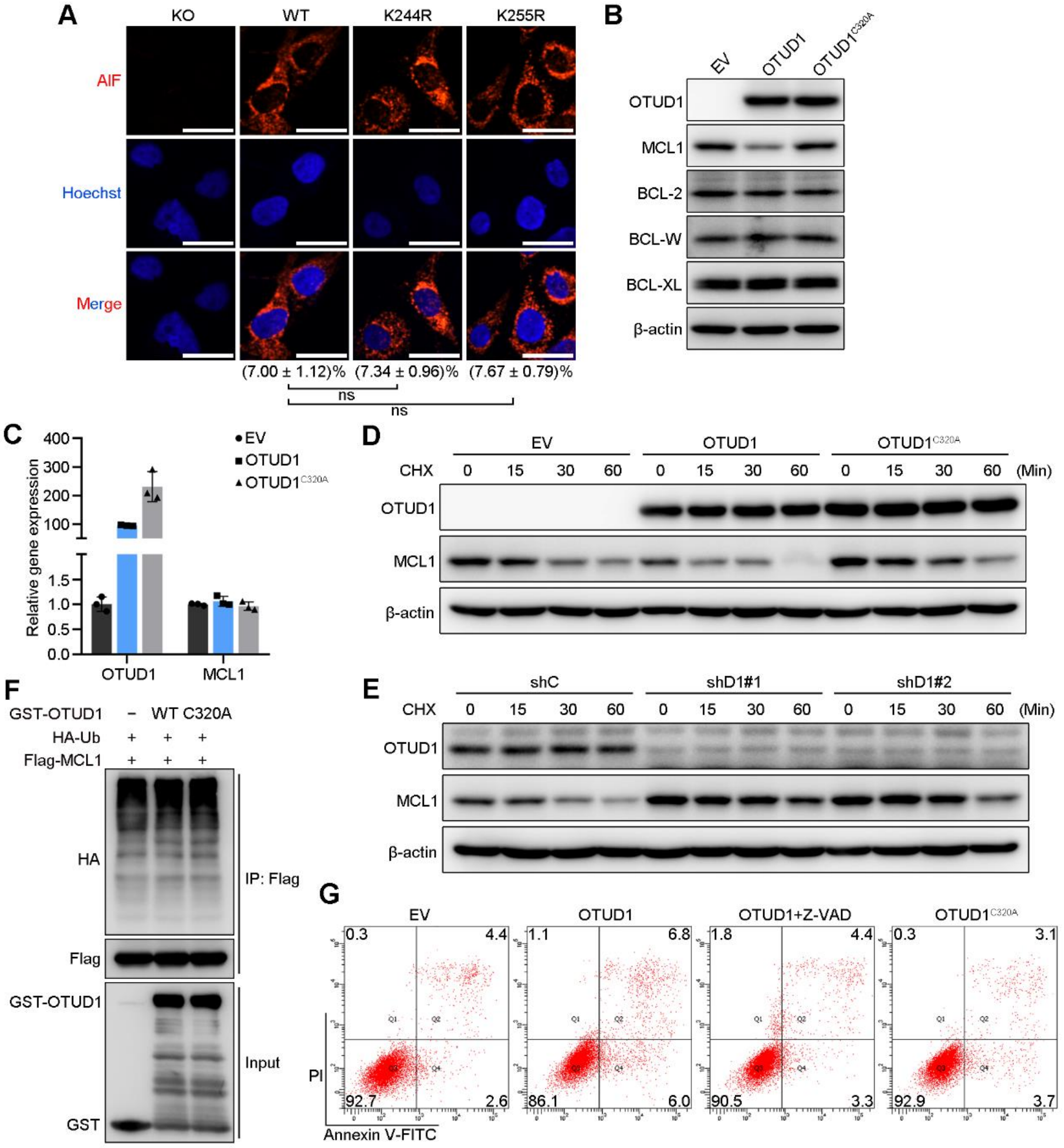

Figure S6. OTUD1 promotes MCL1 degradation. A) Confocal microscopy of the localization of AIF (red) in the indicated cells (KYSE30 background). Nuclei were stained with Hoechst 33342 (blue). Scale bars, 30 µm. Percentages indicate the relative intensity of the AIF signal inside the nucleus compared to the AIF signal of the whole cell. The data are the means ± s.e.m.; $n = 15$. Two-tailed *t* tests, ns: not significant. B,C) IB and qRT-PCR to measure the expression of the indicated proteins or mRNAs in KYSE30 cells expressing EV, OTUD1 or OTUD1^{C320A}. The data are the means ± s.d.; $n = 3$. D,E) IB for MCL1 expression in KYSE30 cells expressing EV, OTUD1 or OTUD1^{C320A} (D) or in KYSE150 cells expressing shC, shD1#1, or shD1#2 shRNAs (E) that subjected to a CHX pulse-chase assay. F) IB to assess MCL1 ubiquitination in an *in vitro* ubiquitination assay. G) Representative images of the apoptotic fraction of *AIF*-ablated KYSE30 cells expressing EV, OTUD1 or OTUD1^{C320A} and treated with or without Z-VAD (50×10^{-6} M) in flow cytometric analysis.

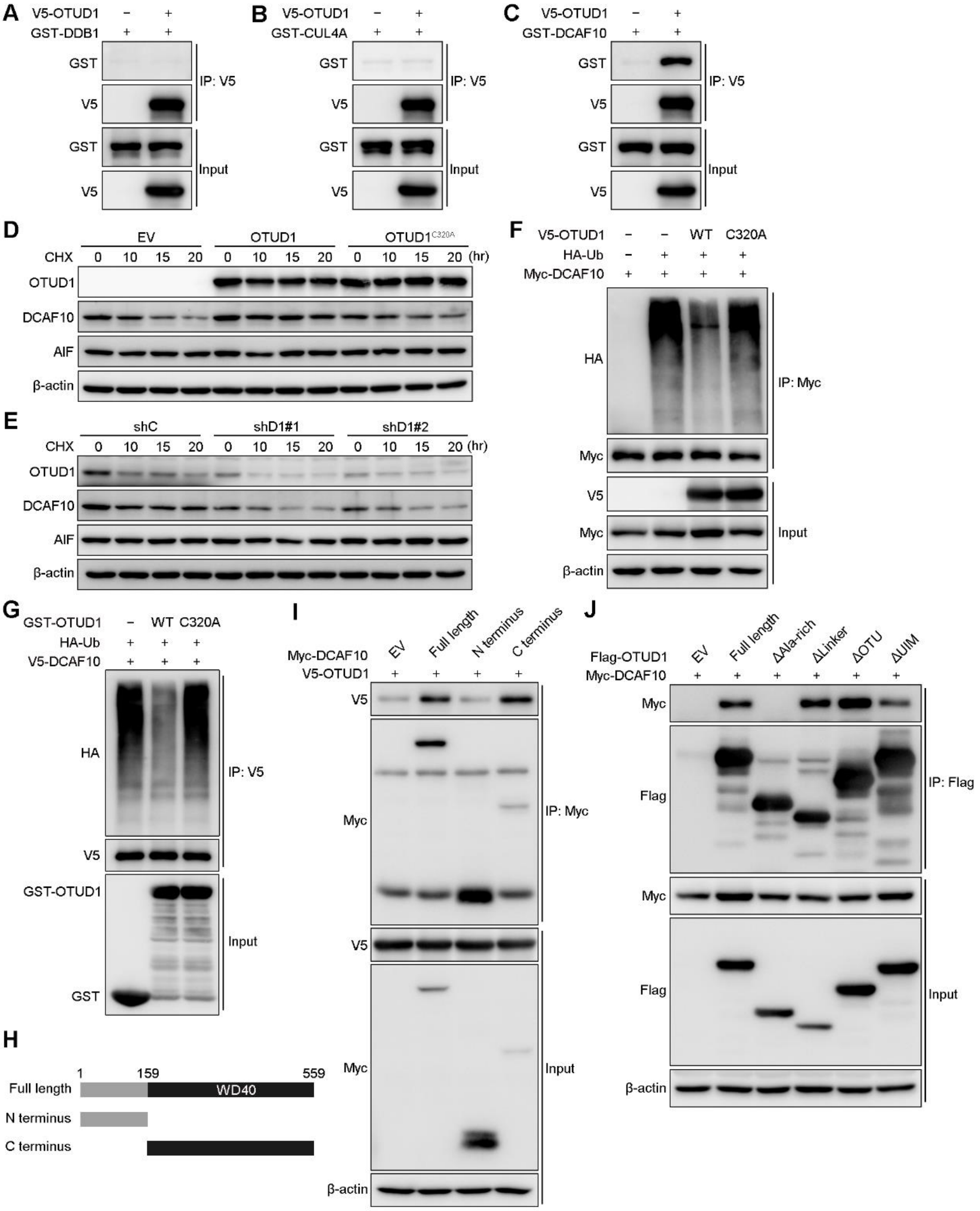

Figure S7. OTUD1 associates with and stabilizes DCAF10. A-C) IB for the indicated proteins in *in vitro* co-IP assays. D,E) IB for the indicated proteins in KYSE30 cells expressing EV, OTUD1 or OTUD1^{C320A} (D) or in KYSE150 cells expressing shC, shD1#1, or shD1#2 shRNAs (E) that subjected to a CHX pulse-chase assay. F,G) IB for DCAF10 ubiquitination in *in vivo* (F) and *in vitro* (G) ubiquitination assays. H, Schematic showing truncated mutants of DCAF10. I,J) IB for the indicated proteins in co-IP assays.

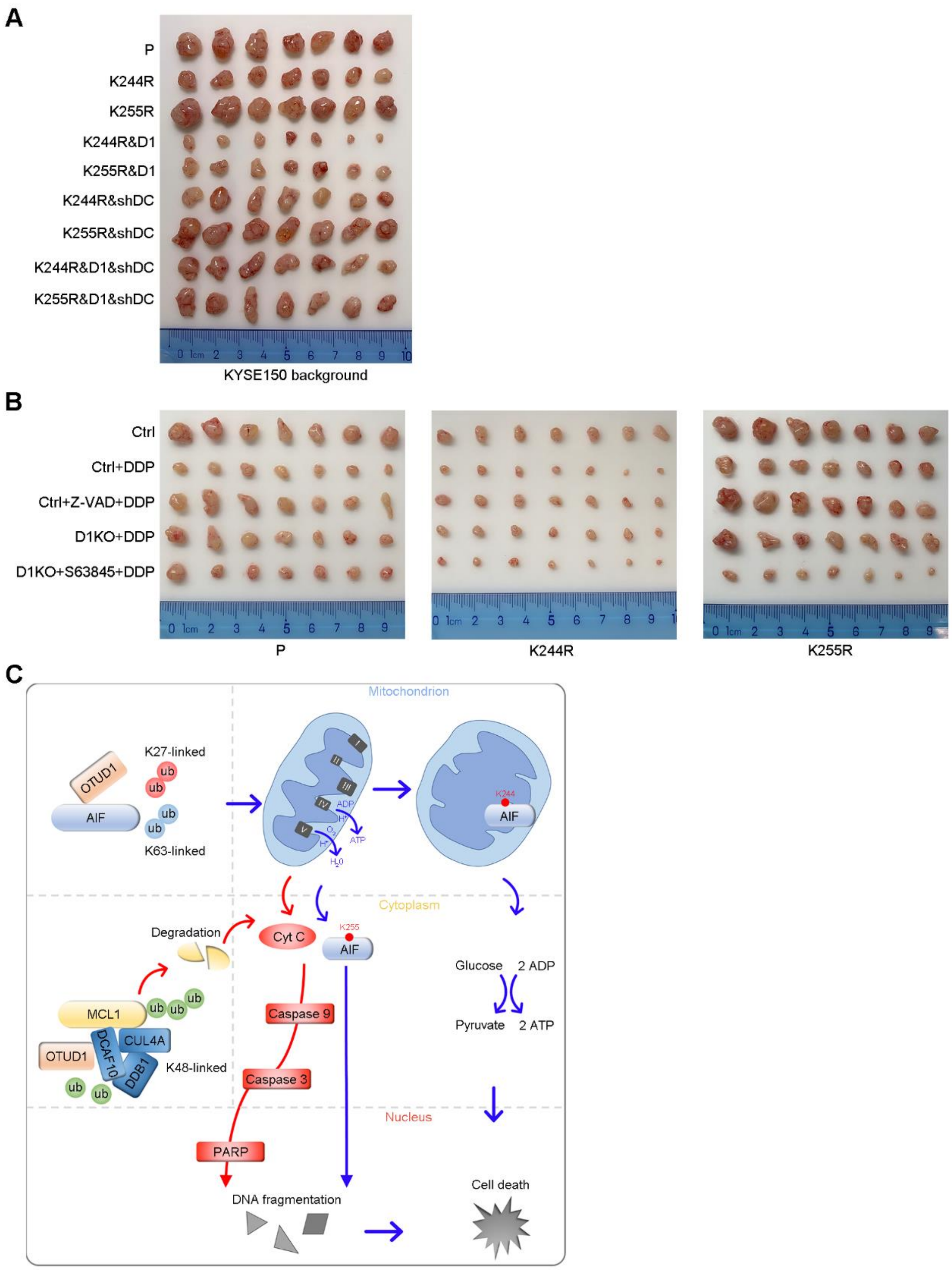

Figure S8. OTUD1 depletion promotes chemoresistance via inhibition of parthanatos and caspase-dependent apoptosis signaling. A) Representative image of xenografts from the indicated cells (KYSE150 background). B) Representative image of xenografts from the indicated cells (KYSE150 background) treated with DDP, Z-VAD, or S63845. C) A schematic model showing that OTUD1 activates both caspase-independent and caspase-dependent apoptotic signaling via the promotion of AIF deubiquitination and MCL1 degradation. In the mitochondrion, OTUD1 deubiquitinates AIF at K244 and K255 and recruits the CUL4A-DDB1 complex via

stabilization of DCAF10 to promote the ubiquitination of MCL1. AIF with deubiquitinated K244 leads to a destructive mitochondrion and compromises OXPHOS to induce cell death. In the cytoplasm, degraded MCL1 promotes the release of cytochrome C (Cyt C) and AIF. The release of Cyt C activates caspase-dependent apoptotic signaling, while AIF with deubiquitinated K255 binds to DNA and induces parthanatos.

Table S1. List of qRT-PCR primer sequences

Primer name	Sequence (5'-3')
GAPDH-F	CCGGGAAACTGTGGCGTGATGG
GAPDH-R	AGGTGGAGGAGTGGGTGTCGCTGTT
UCHL1-F	GACGAATGCCTTTTCCGGTG
UCHL1-R	GACTTCTCCTTGCTCACGCT
SENP8-F	AAGAGGCAGAAGACAGAAGGC
SENP8-R	CCAGCTTGGCGGATCCAATA
SENP6-F	AGCAGTCTGGACCGAAAAGA
SENP6-R	AGGCTCCACTTGTGATTCGG
JOSD2-F	GATCACCGGGAGCTGTAGTC
JOSD2-R	GCGGCCATGATCACATTGAC
ATXN3L-F	CTCTCCCAGTTACCTGCCAA
ATXN3L-R	GCACCATAGTAGTGCCCGTT
A20-F	GCCTACAACCCGCATACAAC
A20-R	GCGATCCTTTCGCAAAGTCC
AMSH-F	TGGAATTGCTCAACCAGGTG
AMSH-R	CTGCCTTCAGCTCTTCTGCT
CEZANNE2-F	GTGATCCCCCTGACGGATTC
CEZANNE2-R	TCAGCTTGGCTTCTAGCGAC
MYSM1-F	GACTGTGGCTTCAACATGCC
MYSM1-R	GGCTTCAACTCAGGTGCTCA
CEZANNE-F	GCCAGCTCCAGCATTGTTTC
CEZANNE-R	CCGATCATGGAAACCCCACA
USPL1-F	CCTTGGAGCGGAATGAGATTT
USPL1-R	CCAGTGGCATTTCCAGTTTAGAT
EIF3S5-F	AGGCTGCACCCAGTCATTTT
EIF3S5-R	CCCAACAGGGTCCCGATAAC
PRPF8-F	TGCCTCATTCATCGTGCAGT
PRPF8-R	GCCTCATCTGCTGTGAACCT
BAP1-F	CAGTGCTTTCAACTCGCCAC
BAP1-R	CGTATGCAGTCAACACGCAG
DESI2-F	GGCTAACCAGTTAGTGGTGCT
DESI2-R	AGGATGGCCACCATAAGCAAA
MPND-F	ACTATTCTGGCAACCCAGGC
MPND-R	CGTAGGCCATCTCCACATCC
SENP1-F	AAGCCCAGACTGAAAAGGGT
SENP1-R	GCTGGTCAGAAAGCGAAAGC
OTUD6B-F	GGAGGAAGCAACTCACCGAA
OTUD6B-R	GCAGCTTTCTTTTCCCGTCTC
SENP7-F	GGCCATCTTCATCCGAAATCA
SENP7-R	CAAAGGGAGAGTCCAGCGT
TRABID-F	TGTACCCAGTGCTTATCCCAA
TRABID-R	TGCTGTGTCCTAGTGTTCAGTT

DESI1-F	CTGCCTCACCAGGGCTTTTC
DESI1-R	ATCCGAAGTGAACGCACAGA
SENP5-F	GATATGGACGACCTGGCGAC
SENP5-R	AGTGGACTTCCAGGTGAATAGG
OTUB2-F	CTTTGGTTTGCGGAGCGG
OTUB2-R	GGATGGCGGTGAACCTTTTG
JOSD1-F	GGGATACGCTGCAAGAGATTT
JOSD1-R	CCATGACGTTAGTGAGGGCA
EIF3H-F	AGGAAGGTACCGGCTCTACT
EIF3H-R	TGCTTCACGGCTGAATCTCC
CSN6-F	TCATCGAGAGCCCCCTCTTT
CSN6-R	TCTGTGGCCAGAGTGTAGGT
VCPIP1-F	AGGCACCTTCGGGATCAAAG
VCPIP1-R	CCCAGTGCATCTGCTACACA
BRCC3-F	TCGTTTGTCTCAACCACGCT
BRCC3-R	AGCAACTGTGCGCATTTCAG
OTUD5-F	TGCGAAAGCATTGCATGGAC
OTUD5-R	GTTCCACTGCAGAAGTACCTG
SENP2-F	GGCTGGTTAGGATTCTCGGC
SENP2-R	GGCAGCATTGTAGAGACTGTTTT
ATXN3-F	GCTGGATGAGGAGGAGAGGA
ATXN3-R	GCTGCTGTAAAAACGTGCGA
PSMD14-F	TCACCCTGGCTTTGGTTGTT
PSMD14-R	CACAGCTCTCTCCGACAAGG
UCHL5-F	GGTTTGCTGAGATCTGTGGC
UCHL5-R	TTTCCATGAGGCACCACTCC
SENP3-F	AGGACATGCCCAAACTTCGT
SENP3-R	TCCCATTTATGGGCTTGGGG
AMSHLP-F	CTGCTATGCCTGACCATACAG
AMSHLP-R	CAGACCTAAAGTAACGTCGTGG
OTUD4-F	AGGAAGCAGGCGGAGAATGGA
OTUD4-R	GCTGGCACAGTGGCTGGTAA
CSN5-F	GCGCCTTTAGGACATACCCA
CSN5-R	CATGAAACTCCCTCGTCCCA
OTUD6A-F	CCCGCTTGCCATTCAACATC
OTUD6A-R	TTCGTCTTGTCGGTCTTGGG
OTULIN-F	CTAACTTTCTGGCACCCGCA
OTULIN-R	GCGGGAATGCTGTTGAATCC
FAM105A-F	TGATGGTAGCTTGTTCCCACT
FAM105A-R	TGTTTCCAAACAGGCAGGGA
OTUB1-F	GAGCACCTCCGACTACCTTG
OTUB1-R	ATGTGGATGTGGTCGCTCTC
YOD1-F	TAAACGTGGTGCTTCTAGTTACG
YOD1-R	CCTTCGACGACATAGTACACAC

PSMD7-F	ACCTGGAAAAAGTCGCCACA
PSMD7-R	ATCAGCGAGGCCAAGTACAC
OTUD3-F	AGAGCCTCCACCAGCACTAA
OTUD3-R	TGCAGCTTCTATTGCACCAAGT
